# Systematic Review and Meta-analysis of Anti-interferon Auto-antibodies in Infectious Diseases

**DOI:** 10.1007/s10875-026-02038-6

**Published:** 2026-06-09

**Authors:** Robert Hill, Akish Luintel, Ernest Mutengesa, Douglas L Fink

**Affiliations:** 1 St Mary’s Hospital, London, W2 1NY UK; 2 Department of Clinical Microbiology, Infection Division, University College London Hospital, London, NW1 2BU UK; 3 Institute of Health Informatics, University College London, London, NW1 2DA UK; 4 Department of Infectious Diseases, Royal Free Hospital, Royal Free London NHS Foundation Trust, London, NW3 2QG UK; 5 Institute of Infection, Immunity and Transplantation, University College London, London, NW3 2PP UK

**Keywords:** Anti-interferon auto-antibodies, SARS-COV-2, COVID-19, Infectious diseases

## Abstract

**Purpose:**

To perform a systematic review and meta-analysis of prevalence and function of anti-interferon auto-antibodies in acute infectious diseases.

**Methods:**

We performed a search on the following electronic bibliographic databases: Medline, Embase, Web of Science and Cochrane. Eligible studies generated a systematic review and random effects model meta-analysis of pooled seroprevalence and neutralisation status of anti-interferon auto-antibodies in acute infectious diseases.

**Results:**

Thirty-seven studies including 12,629 individuals with SARS-CoV-2 infection were analysed. There are insufficient data for meta-analyses of auto-antibodies in non-SARS-CoV-2 infectious diseases, with crude seroprevalence of auto-antibodies varying widely (0.0–77.0%). The pooled seroprevalence of anti-IFNɑ and/or anti-IFN⍵ auto-antibodies in individuals with SARS-CoV-2 infection was 14% (95%CI 9–18%, *I*^2^ = 92%, $\:\tau\:$^2^ = 0.0151, *p* < 0.01). Pooled prevalence of neutralising auto-antibodies were slightly lower (12%; 95%CI 8–17%, *I*^2^ = 77%, $\:\tau\:$^2^ = 0.0027, *p* < 0.01). The high heterogeneity may reflect divergent SARS-CoV-2 disease severity across studies, and methodological diversity for measurement and definition of auto-antibody binding and neutralisation. Pooled seroprevalence for anti-IFNɑ auto-antibodies in uninfected healthy controls were < 1% (95%CI 0–1%, *I*^2^ = 90%, $\:\tau\:$^2^ = 0.0028, *p* < 0.01).

**Conclusion:**

Anti-interferon auto-antibodies may impair immune responses to diverse infections leading to life-threatening disease. These auto-antibodies have not been studied at all in most infectious diseases, most notably for bacterial infection. This study illustrates the urgent need to standardise methodology and reporting of auto-antibodies in the setting of infectious diseases so that the immunopathology and translational impact of these novel biomarkers can be realised.

**Supplementary Information:**

The online version contains supplementary material available at 10.1007/s10875-026-02038-6.

## Introduction

Interferon (IFN) proteins are abundant cytokines central to diverse anti-pathogen immune responses. There are three classes of interferon (IFN): type 1 IFN (T1IFN), type 2 (T2IFN) and type 3 (T3IFN) which each signal though a distinct heterodimeric receptor to induce anti-pathogen gene expression [1]. In humans there are 13 partially homologous IFNα subtypes, a single IFNβ, as well as several other poorly defined single gene products including IFNε, IFNτ, IFNκ, IFNω, IFNδ and IFNζ [2]. IFN-γ is the only member of the T2IFN class while four T3IFN subtypes are described (IFN-λ1–4).

Since the 1980s pathogenic auto-antibodies (auto-abs) neutralising IFN, which antagonise downstream immune signalling, have been recognised to underlie severe or fatal clinical infectious disease phenotypes, such as disseminated shingles [3, 4]. This form of immunodeficiency, until recently only described in case reports or series, was previously considered rare. During the 2020 SARS-CoV-2 pandemic large cohort studies emerged for the first time suggesting that anti-IFN auto-abs are common drivers of poor outcomes from infectious diseases due to their presence in up to 20% of patients with life-threatening SARS-CoV-2 infection [5–7]. These unexpected findings stimulated a proliferation of similar cohort studies across infectious diseases settings. However, estimates for the prevalence of anti-T1IFN auto-abs in patients with life-threatening respiratory virus infections, including SARS-CoV-2, Middle East Respiratory Syndrome (MERS) and influenza, vary widely between 5 and 33% [6, 8, 9]. Cohort studies of anti-T1IFN auto-abs in the context of non-respiratory infections are less common, but suggest prevalence of up to 40% of individuals with West Nile virus (WNV) encephalitis [10] Chronic infectious diseases are also understudied however estimates suggest that 16.7% of people with chronic hepatitis B virus infection [11]. The clinical significance of auto-abs against T2IFN and T3IFN are less well characterised although the former are associated with severe mycobacterial infections [12, 13].

There are no standardised assays for detection of anti-IFN auto-abs or measurement of function. Across studies, there is heterogeneity of methodology used to measure anti-IFN auto-ab binding and function, which has never been formally reviewed. Neutralising anti-IFN auto-abs are more strongly associated with poor outcomes compared to non-neutralising auto-abs [14]. In most studies, characterisation of anti-IFN auto-ab function is limited to assays measuring neutralisation of IFN signalling in vitro using reporter cell lines [15]. Many studies do not report auto-ab function. Since publication in 2023 of two systematic reviews of anti-T1IFN auto-abs in SARS-CoV-2 [16, 17], many more recent studies of anti-IFN auto-abs have followed in the setting of COVID and other infections which have never been subject to meta-analysis or systematic review. Importantly, there are no systematic review or meta-analysis studies for anti-IFN auto-abs in infectious diseases other than SARS-CoV-2.

For the first time, this study sought to undertake systematic review and provide meta-analysis estimates for the seroprevalence of binding and neutralising auto-ab against all IFN sub-types in all acute infectious diseases.

## Methods

### Search Strategy and Selection Criteria

Included study designs were human observational studies that reported data for human participants over the age of 18 with a confirmed infectious disease with any measure of prevalence of auto-abs against any IFN sub-type during the infection episode. Studies published between January 1 1946 to February 24th 2025 were included, including peer-reviewed articles, letters and pre-prints. Studies were not required to include a control population. Community-based, hospital-based and mixed studies were all included. No restriction was placed on sample size for study selection but case reports and case series were excluded from analysis (Supplementary Data 1).

Infectious diseases are defined as laboratory confirmed infections by a recognised pathogenic microorganism including those which are transmitted human-to-human or animal-to-human (zoonotic). Acute infectious diseases are defined by the presence of symptoms at the time of study inclusion suggesting active disease. Chronic viral infectious disease (such as HIV or chronic hepatitis B virus infection) were not included as the majority of individuals are asymptomatic at the time of diagnosis. Past infectious diseases, or presumed post-acute sequelae of infectious diseases, including long COVID, were not included.

**Fig. 1:**
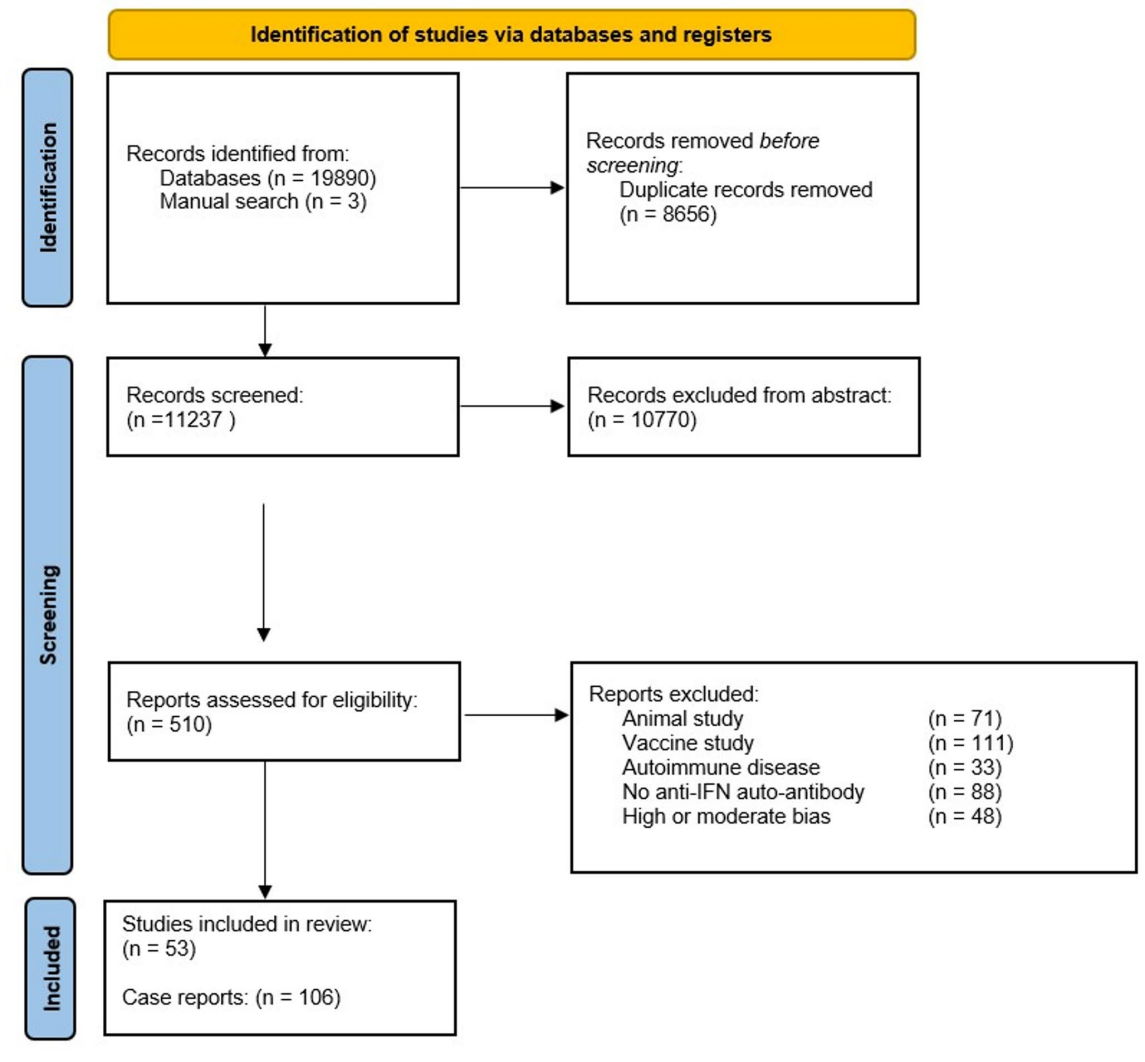
PRISMA flow diagram

A systematic search was conducted using MEDLINE (Ovid), MEDLINE (PubMed], Embase (Ovid), Web of Science and Cochrane Library databases. The original search was run on 10/05/2023 and updated on 24/02/2025. Two independent reviewers (R.H. and E.M.) screened studies, first by title and abstract, and then screening remaining full texts for potentially eligible articles (Fig. 1). Any discrepancies were resolved by a third independent reviewer (D.L.F.). This systematic review and meta-analysis was conducted and is reported according to Preferred Reporting Items for Systematic Reviews and Meta-Analysis (PRISMA). The study is registered on PROSPERO (registration number CRD42023448147). The search was conducted according to a study protocol (Supplementary Data 2).

### Data Analysis

Data were extracted by one researcher and reviewed by a second. Any discrepancies were resolved by consensus between the two or by a third party (D.L.F.). Studies that were duplicates, unavailable as full text or abstract only were excluded.

The primary outcomes are anti-IFN auto-ab prevalence and prevalence of neutralising anti-IFN auto-ab. Any laboratory assay could be used to detect auto-ab and measure neutralisation. For studies using multiple methods to estimate anti-IFN auto-abs, each estimate was included independently. Assays using either plasma and/or serum samples, including purified immunoglobulin, were included. Auto-ab against any IFN subtype were included and prevalence estimated independently where possible. Additionally, estimates were made for combined prevalence of auto-ab against IFNɑ and IFN⍵ which are the two most abundant T1IFNs. Data extracted from each article included author, study title, the number of participants, infectious disease, auto-ab detection method, sub-type of anti-IFN auto-ab detected, anti-IFN auto-ab prevalence, anti-IFN auto-ab neutralisation status (defined by each individual study), 28-day mortality, infectious disease severity. For studies using Gyrolabs immunoassays where no auto-ab detection cut-off calculation was provided, only prevalence data for a ‘high’ Gyrolabs cut-off (> 100 Gyros arbitrary units) were used for prevalence estimates. For in vitro IFN neutralisation assays where multiple concentrations of IFN were used, prevalence estimates of IFN neutralisation were based on the lower dose of IFN which are likely closer to physiological levels in vivo. Where reported, data were also extracted for healthy controls without infectious diseases. Studies that did not provide methodology for defining cut-offs for detection of auto-ab were considered at high risk of bias.

Secondary outcomes included 28-day mortality between participants with and without anti-IFN auto-ab, and anti-IFN auto-ab in chronic infection (duration more than 6 months), and auto-ab prevalence stratified by infectious disease severity, categorised as ‘community’, ‘hospitalisation’ or ‘intensive care unit’. Infectious disease severity was categorised If a study did not explicitly differentiate between ‘hospitalisation’ or ‘intensive care unit’, data were reported as ‘hospitalisation’.

Risk of bias was assessed using the Joanna Briggs Critical Appraisal Checklist for Prevalence Studies. Two independent reviewers (R.H. and E.M.) performed component analyses and a third independent reviewer (D.L.F.) resolved disagreements. Component scores were synthesised to generate a risk of bias assessment (low, moderate, high) determined using a consensus decision rule agreed by all authors (Supplementary Data 3). Studies meeting 8–9 components of the JBI checklist were considered low risk of bias, meeting 5–7 components considered moderate risk of bias and meeting 4 or less components high risk of bias [18]. Only studies with a low risk of bias were included in the final meta-analyses.

### Statistical Analysis

Data on anti-IFN auto-Abs are summarised descriptively. Owing to the significant heterogeneity between the studies, the prevalence of anti-IFN auto-abs and the 95% CI were calculated by applying a random-effects model (REML methodology) [19], using a Freeman-Turkey double arcsine transformation [20]. This was undertaken on the “metaprop” package on R studio. The heterogeneity was quantified both in absolute terms (range of individual study estimates) and as a proportion of total variation (I2), and tested across predefined subgroups. A sensitivity analysis was conducted stratifying prevalence of each cohort by infectious disease severity.

### Patient Consent Statement

No formal ethics approval was required for this study as all data analysed was from existing published studies and no transfer of data was involved.

## Results

### Literature Search

The screening strategy identified 11,237 studies once duplicate records had been removed. After title and abstract screening, 507 full-text studies were assessed for eligibility. Reasons for exclusion are summarised in Fig. 1. Overall, 53 studies reported data for 15,148 participants in the context of 12 infectious diseases for whom descriptive summary analyses were performed (Table 1). After risk of bias assessment, meta-analysis was only possible for anti-T1IFN auto-abs prevalence during SARS-CoV-2 infection. All studies of other infectious diseases and all data for anti-IFN auto-abs against non-T1IFN sub-types were not included in meta-analyses (Table 1).

**Table 1:** Crude seroprevalence of anti-interferon auto-antibodies organised by pathogen

Pathogen	Total studies	Total participants	Anti-IFNα and/or IFNω auto-Abs	Anti-IFNα auto-Abs	Anti-IFNβ auto-Abs	Anti-IFNω auto-Abs	Anti-IFNγ auto-Abs	Anti-IFNλ auto-Abs
Viruses								
SARS-CoV-2	37	12,629	2067/15,787 (13.1%)	1378/ 16,755 (8.2%)	69/ 4183 (1.6%)	895/ 11,643 (7.7%)	23/ 224 (10.3%)	51/1038 (4.9%)
Non-COVID pneumonia^a^	5	580	13/279 (4.7%)	6/205 (2.9%)	11/151 (7.3%)	-	18/181 (9.9%)	-
West Nile virus	2	516	294/882 (33.3%)	283/957 (30.0%)	29/441 (6.6%)	247/957 (25.8%)	-	-
Tick-borne Encephalitis Virus	1	441	-	3/441 (0.7%)	1/441 (0.2%)	4/441 (0.9%)	-	-
Ross River virus	1	96	-	1/96 (1.0%)	0/96 (0%)	0/96 (0%)	-	-
Middle East respiratory syndrome coronavirus	1	62	13/62 (30.0%)	10/62 (16.1%)	3/62 (4.8%)	9/62 (14.5%)	-	-
Usutu virus	1	34	-	1/34 (2.9%)	1/34 (2.9%)	1/34 (2.9%)	-	-
Powassan virus	1	3	-	0/2 (0.0%)	0/2 (0/0%)	1/3 (33.3%)	-	-
Mycobacteria								
*Mycobacterium tuberculosis*	2	389	-	-	-	-	42/389 (10.8%)	-
Non-tuberculosis mycobacteria	6	259	-	-	-	-	198/259 (76.4%)	-
Bacteria								
*Escherichia coli* ^b^	1	78	-	3/78 (3.8%)	-	-	-	-
Fungi								
*Talaromyces marneffei*	2	100	-	-	-	-	77/100 (77.0%)	-

Crude seroprevalence for each pathogen or syndrome was calculated by dividing the total number of detectable auto-antibody results by the number of auto-antibody tests performed in the study populations across all studies

Abbreviations: SARS-CoV-2, Severe Acute Respiratory Syndrome Coronavirus 2; COVID, Coronavirus disease 2019

^a^Non-COVID pneumonia pathogens: influenza, rhinovirus, seasonal coronavirus, parainfluenza, metapneumovirus, bacterial pneumonia, non-covid viral pneumonia

^b^*Escherichia coli* (*E. coli*) bacteraemia (*n* = 77) and *E.coli* meningitis (*n* = 1)

### Prevalence Estimates for SARS-CoV-2 Infection

Meta-analysis was undertaken for 37 studies including 12,629 participants with laboratory confirmed SARS-CoV-2 infection (Table 2) [5–7, 13, 21–53].

**Table 2:** Crude seroprevalence of anti-interferon auto-antibody in patients with SARS-CoV-2 infection stratified by auto-antibody neutralisation status and level of care

SARS-CoV-2 infection level of care	Total participants	Neutralisation status	Auto-Ab					
			IFNα and/or IFNω	IFNα	IFNβ	IFNω	IFNγ	IFNλ
Community	1770	Neut	17/1639 (1.0%)	5/1639 (0.3%)	-	13/1639 (0.8%)	-	-
		Non-neut	10/1683 (0.6%)	0/87 (0.0%)	-		-	-
Hospitalised (including ICU)	10,379	Neut	783/6357 (12.3%)	747/8612 (8.7%)	19/2540 (0.7%)	686/7520 (9.1%)	-	-
		Non-neut	566/6108 (9.3%)	602/4625 (13.0%)	49/146 (3.3%)	199/2365 (8.4%)	23/224 (10.3%)	51/506 (10.1%)
ICU	6833	Neut	654/5165 (12.7%)	582/6065 (9.6%)	14/1912 (0.7%)	567/5898 (9.6%)	-	-
		Non-neut	531/5328 (10.0%)	262/2063 (12.7%)	20/1008 (2.0%)	184/1827 (10.1%)	-	-

Crude seroprevalence for each pathogen or syndrome was calculated by dividing the total number of detectable auto-antibody results by the number of auto-antibody tests performed in the study populations across all studies. Neutralisation status was defined by individual study criteria

Abbreviations: Auto-Ab, auto-antibody; Neut, neutralising; Non-neut, non-neutralising; ICU, intensive care unit

**Fig. 2:**
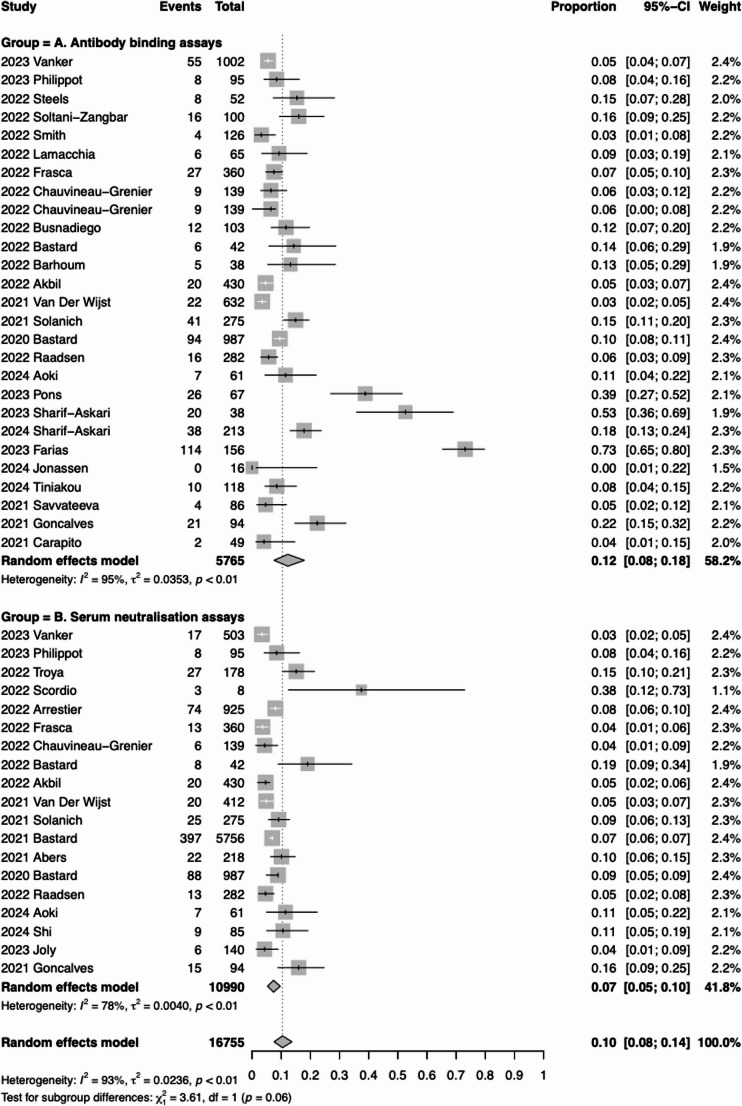
Forest plot of anti-IFN$\:\alpha\:$ seroprevalence in all patients. Group **A** illustrates studies using auto-ab detection by auto-ab binding, and group **B** detection by IFN neutralisation assays. 95% prediction intervals shown

**Fig. 3:**
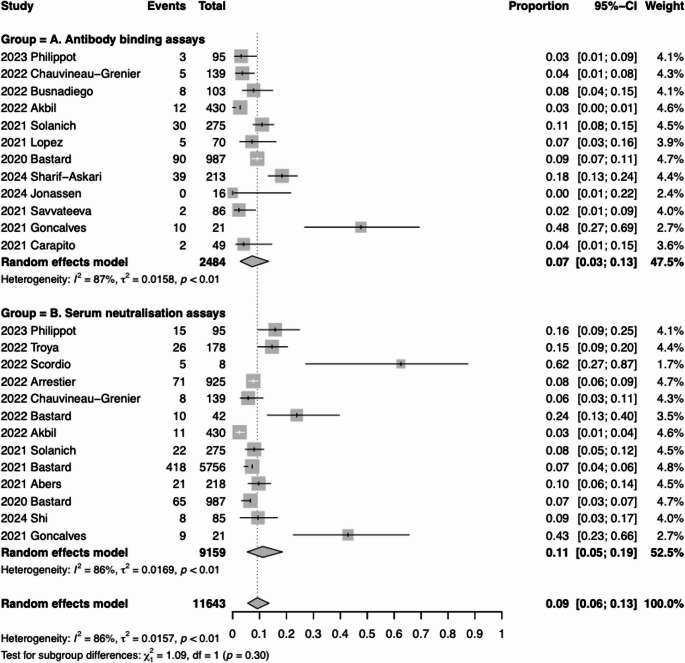
Forest plot of anti- IFN$\:\omega\:$ seroprevalence in all patients. Group **A** illustrates studies using auto-ab detection by auto-ab binding, and group **B** detection by IFN neutralisation assays. 95% prediction intervals shown

**Fig. 4:**
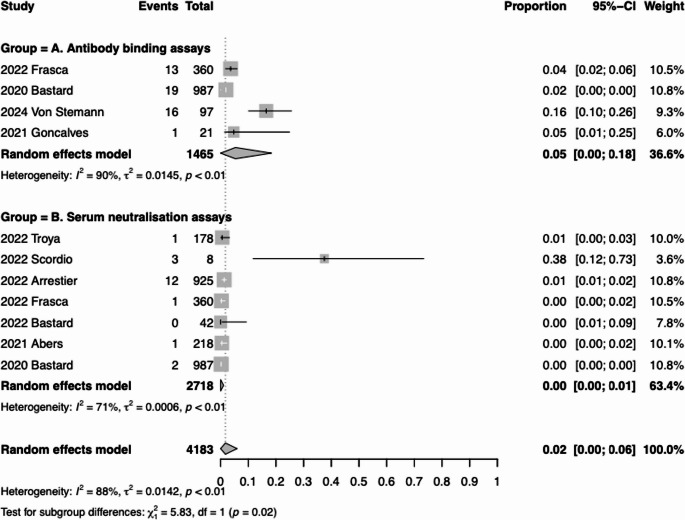
Forest plot of anti-IFN$\:\beta\:$ seroprevalence in all patients. Group **A** illustrates studies using auto-ab detection by auto-ab binding, and group **B** detection by IFN neutralisation assays. 95% prediction intervals shown

Anti-IFN$\:\alpha\:$ auto-abs were tested in 16,755 samples. Prevalence of auto-abs against IFN$\:\alpha\:$ in individuals with SARS-CoV-2 infection, including all severities of disease, was 12% (95%CI 8–18%, I^2^ = 95%; Fig. 2), with IFN$\:\alpha\:$-neutralising auto-abs present in 10% (95%CI 8–14%, I^2^ = 78%; Fig. 2). 26/37 studies tested only the IFN$\:\alpha\:$2 subtype and a further 7/37 studies did not state which IFN$\:\alpha\:$ subtype was tested. Anti-IFN⍵ auto-abs were tested in 11,643 samples. Prevalence of auto-abs against IFN⍵ at any disease severity was 7% (95%CI 3–13%, I^2^ = 87%; Fig. 3) with IFN⍵ neutralising auto-abs present in 11% (95%CI 5–19%, I^2^ = 86%; Fig. 3). Anti-IFNβ auto-abs were tested in 2031 samples. Prevalence of auto-abs against IFNβ was 2% (95%CI 0–6%, I^2^ = 88%; Fig. 4) with IFNβ neutralising auto-abs present in < 1% (95%CI 0–1%, I^2^ = 71%). 2/34 studies assessed anti-IFNγ auto-abs in SARS-CoV-2 infection and 1/34 studies assessed anti-IFNλ auto-abs, which were not subjected to meta-analysis. Auto-abs against other IFN sub-types were not measured.

**Fig. 5:**
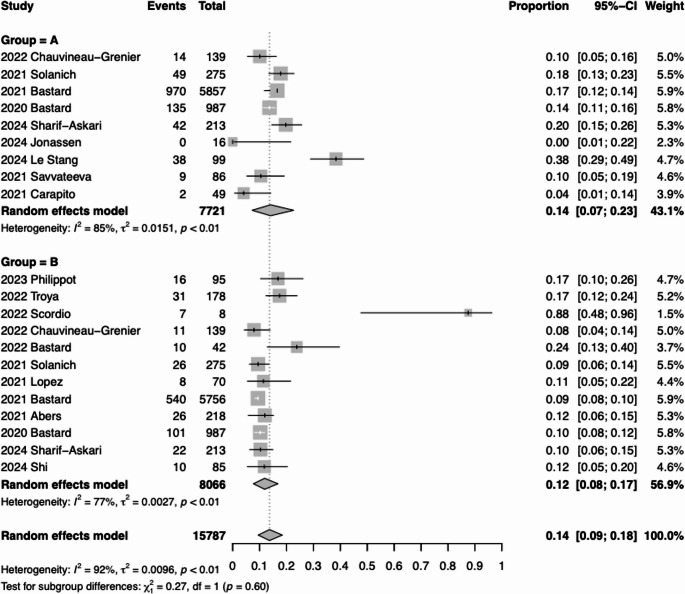
Forest plot of anti-IFN$\:\alpha\:$ and/or anti-IFN$\:\varpi\:$ seroprevalence in all patients. Group **A** illustrates studies using auto-ab detection by auto-ab binding, and group **B** detection by IFN neutralisation assays. 95% prediction intervals shown

Overall, anti-IFN$\:\alpha\:$ and/or anti-IFN⍵ auto-abs were measured 15,787 times in the population studied. For individuals diagnosed with SARS-CoV-2 infection of any disease severity, prevalence of anti-IFN$\:\alpha\:$ and/or anti-IFN⍵ auto-Abs was 14% (95%CI 7–23%, I^2^ = 85%; Fig. 5). Prevalence of serum or plasma neutralisation of IFN$\:\alpha\:$ and/or IFN⍵ was lower at 12% (95%CI 8–17%, I^2^ = 77%; Fig. 5).

**Fig. 6:**
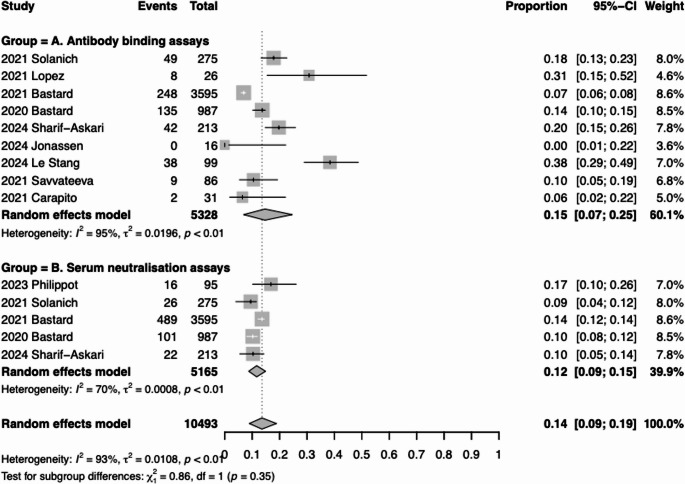
Forest plot of anti-IFN$\:\alpha\:$ and/or anti-IFN$\:\varpi\:$ seroprevalence in patients with severe COVID-19. Group **A** illustrates studies using auto-ab detection by auto-ab binding, and group **B** detection by IFN neutralisation assays. 95% prediction intervals shown

The majority of studies included patients hospitalised with SARS-CoV-2 infection (37/37 (100%) studies; 10379/12629 (82.2%) participants). 19/37 studies of patients with SARS-CoV-2 infection included patients admitted to an intensive care unit (ICU; 19/37 (51%) studies; 6833/12629 (54.1%) participants). 4/37 (11%) studies provided 28-day mortality data which were not suitable for meta-analysis. Prevalence of anti-IFN$\:\alpha\:$ and/or anti-IFN⍵ auto-abs in patients with SARS-CoV-2 infection requiring ICU admission was 15% (95%CI 7–25%, I^2^ = 95%; Fig. 6; Table 2) with neutralising auto-abs present in 12% (95%CI 9–15%, I^2^ = 70%). Prevalence of anti-IFN$\:\alpha\:$ auto-abs in patients with SARS-CoV-2 infection requiring ICU admission was 12% (95%CI 9–16%, I^2^ = 78%; Supplementary data 4) with IFN$\:\alpha\:$ neutralising auto-abs present in 9% (95%CI 7–11%, I^2^ = 49%; Supplementary data 4). For healthy control populations without SARS-CoV-2 infection, prevalence of anti-IFN$\:\alpha\:$ auto-abs was measured in 17,302 samples and reported as < 1% whether measured by binding or neutralisation assays (95%CI 0–1%, I^2^ = 90%; Supplementary data 5). Hospitalised patients had higher prevalence of all tested anti-IFN auto-Abs than patients in the community at 11% (95% CI 8–15%, I2 = 93%; Supplementary data 6 group A) vs. < 1% (95%CI 0–0%, I2 = 0%; Supplementary data 6 group B).

Most studies (32/37, 86%) reported methodology for deriving cut-offs for auto-ab detection and the remaining studies used arbitrary positivity cut-offs for assays. The most common method of auto-ab detection was ELISA in 22/37 (59%) studies (Supplementary Data 7). 17/37 (46%) used at least two methods for measuring anti-IFN𝛼 auto-Ab prevalence using at least one form of binding assay and one form of IFN neutralisation assay (Supplementary Data 8), and 1/37 studies used two binding assays and one neutralisation assay.

### Prevalence Estimates for Other Infectious Diseases

Eighteen studies tested for anti-IFN auto-abs in non-SARS-CoV-2 infectious diseases (Table 1) [8–10, 39, 50, 54–66]. Six studies reported prevalence of anti-IFNγ auto-abs in mycobacterial infections (Tables 1 and 3) [54–59]. In participants with *Mycobacterium tuberculosis (M.tb)* infection, anti-IFNγ auto-abs were detected in 10.8% (42/389) but all samples were non-neutralising. All of these studies were thought to be at high risk of bias as non-representative of the adult population with mycobacterial infection.

**Table 3:** Crude seroprevalence of anti-interferon auto-antibody in patients with mycobacterial infection stratified by auto-antibody neutralisation status and pathogen

Mycobacteria	Total studies	Total participants	Neutralisation status	Auto-Ab				
				IFNα	IFNβ	IFNω	IFNγ	IFNλ
*Mycobacterium tuberculosis*	2	389	Neut	-	-	-	-	-
			Non-neut	-	-	-	42/389 (10.8%)	-
Disseminated non-tuberculous mycobacteria	6	259	Neut	-	-	-	156/207 (75.4%)	-
			Non-neut	-	-	-	42/52 (80.8%)	-

Crude seroprevalence for each pathogen or syndrome was calculated by dividing the total number of detectable auto-antibody results by the number of auto-antibody tests performed in the study populations across all studies. Neutralisation status was defined by individual study criteria

Abbreviations: Auto-Ab, auto-antibody; Neut, neutralising; Non-neut, non-neutralising

There is no universal definition of disseminated non-tuberculous mycobacteria. In these studies it represents disease in more than one tissue

## Discussion

Our study is the first systematic review and meta-analysis of anti-IFN auto-ab that encompasses SARS-CoV-2 infection, non-SARS-CoV-2 infectious diseases and the healthy general population. Since studies early in the pandemic highlighted the impact of anti-IFN auto-ab on COVID outcomes, there has been a proliferation of studies measuring anti-IFN auto-ab in different cohorts of diverse infectious diseases. Most of these studies post-date the two published systematic reviews reporting prevalence of anti-IFN auto-ab during SARS-COV-2 infection [16, 17].

Our pooled seroprevalence estimates are similar to these systematic reviews for individuals with SARS-CoV-2 infection but should be interpreted with caution for true seroprevalence owing to high heterogeneity between studies. However, even at the lower border of seroprevalence estimates (5%) the impact of these auto-abs suggests clinical relevance at a population level. In our sensitivity analysis of ICU patients with presumed severe COVID only, we also found high heterogeneity as compared to the overall meta-analysis population encompassing all disease severities. This may suggest disease severity or phenotypes across different studies did not explain the heterogeneity detected in our analysis. Although we cannot exclude the possibility that the ICU populations between studies were significantly different also, with participant-level reporting of COVID severity missing from all studies. We did not mandate uninfected control populations as inclusion criteria for studies in our analysis but participant-level demographic and clinical information is also missing for most of the studies that did report auto-ab prevalence in the general population. It will be important for future studies to undertake comparative studies in populations matched to the infectious disease groups.

Anti-IFN auto-ab testing remains predominantly a research tool. We identified extraordinary diversity and inconsistency of techniques used to measure and define cut-offs for auto-ab binding and function (Supplementary Tables 1 and 2). Many studies did not report methodology for definition of auto-ab detection cut-offs at all which has also been highlighted in reviews of autoreactivity in post-COVID syndromes [67]. This emerging field will be greatly aided by standardisation of assays and sharing of serum control samples. Importantly there are no studies reporting seroprevalence and function of auto-ab against the full diversity of T1 and T3 IFNs. This reflects the relatively limited understanding of the biology of these proteins in infectious diseases in all settings, in particular non-alpha and non-beta T1 IFNs (including IFN⍵, IFNε, IFNΚ, IFNδ, IFNθ and IFNτ) and all T3IFNs. T2IFN is structurally and functionally distinct from T1 and T3IFNs, and it remains unknown whether the underlying immunopathology driving auto-ab against IFN proteins is sub-type specific; anti-IFNγ appear rare in setting of clinical autoimmunity syndromes where anti-T1IFNs are more common [68]. Only one study reports auto-ab against T3IFN in patients with COVID and two studies for auto-ab against T2IFN (Supplementary data 9). Beyond IFN proteins, auto-ab against diverse cytokines have been reported in small studies in highly selected populations which warrant reproduction in representative cohorts of infectious diseases [15, 69]. In all studies, the functional characterisation of auto-ab is often limited to neutralisation of cognate immune molecules. The roles of anti-IFN and other anti-cytokine auto-ab in vivo are likely to extend beyond this [70, 71].

Despite the centrality of IFN signalling to immune responses to infection, including non-viral infections, we found data reporting anti-IFN auto-ab prevalence for most infectious diseases although there are many case reports (Supplementary data 10). SARS-CoV-2 is the only pathogen providing more than two studies in this setting with low risk of bias; only two other specific pathogens (West Nile virus and *Mycobacterium tuberculosis*) had two or more dedicated studies but with moderate or high risk of bias [10, 54–59, 62]. Crude seroprevalence estimates varied widely across infectious diseases (0.0–77.0%). Of note only a single study, with only 78 participants, reported on anti-IFN auto-ab in cosmopolitan bacterial infection (*Escherichia coli*) [61]. Excess mortality from bacterial infections is commonly seen in older adults and the crude seroprevalence of anti-IFN auto-abs is thought to increase with age [6]. The impact of anti-IFN auto-abs, and more broadly anti-cytokine auto-abs, in all-cause infectious diseases in older adults warrants urgent attention. Studies are also missing for anti-IFN auto-ab prevalence and clinical associations in the post pandemic, post vaccination era of SARS-CoV-2. Owing to missing demographic and clinical data, our meta-analysis was unable to report seroprevalence by age or sex or vaccine status stratification, or estimates for association with clinical outcomes including mortality.

The underlying aetiology and immunopathogenesis mechanisms of anti-IFN auto-abs remain incompletely understood [15]. The potential utility of anti-cytokine auto-abs as biomarkers for risk stratification has been suggested but without better quality data it is hard to support this. There also remains uncertainty regarding the physiological role of these auto-ab in vivo. For example, the protective effect of anti-cytokine auto-ab in localised mucosal settings is also under-studied. In a single study, high levels of anti-IFN auto-abs in mucosal samples during SARS-CoV-2 infection were associated with more mild disease suggesting an anti-inflammatory immune regulatory role for anti-IFN auto-abs at mucosal surfaces [72]. There are no studies of these auto-ab in mucosal or other tissue samples in other infectious diseases. This exciting field remains in its relative infancy.

## Conclusion

This study is the largest systematic review and meta-analysis of anti-IFN auto-abs in infectious diseases. Consistent with smaller reviews, our pooled estimates suggest high levels of neutralising and non-neutralising anti-T1IFN auto-ab in individuals diagnosed with SARS-CoV-2 infection early in the pandemic prior to vaccination. We found significant heterogeneity between studies which may reflect the lack of standardisation across auto-ab testing methodologies. Importantly there is a scarcity of representative data across many non-SARS-CoV-2 infectious diseases and for auto-ab against other IFN sub-types and cytokines. Future studies are required, with standardised protocols, to help realise the translational potential of these novel auto-ab biomarkers in infectious diseases, and to support study of underlying immunopathology.

## Supplementary Information

Below is the link to the electronic supplementary material.

Supplementary Material 1

## Data Availability

No datasets were generated or analysed during the current study.
