# Supplementary material for: Systematic Review and Meta-analysis of Anti-interferon Auto-antibodies in Infectious Diseases
Source: J Clin Immunol. 2026 Jun 9;46(1):81. doi: 10.1007/s10875-026-02038-6 (PMC13473239; doi:10.1007/s10875-026-02038-6)
Supplement: Supplementary file 1 [file 10875_2026_2038_MOESM1_ESM.docx]

[Supplementary data 1: Search strategy	2](https://docs.google.com/document/d/17LwDdoGhF9zScsCQCgruj1KYc0ySs53nuXKCO7MPmNA/edit?tab=t.0#heading=h.j5m35gw0bjd5)

[Supplementary data 2: Prospero form	3](https://docs.google.com/document/d/17LwDdoGhF9zScsCQCgruj1KYc0ySs53nuXKCO7MPmNA/edit?tab=t.0#heading=h.z16ywaslb1y2)

[Supplementary data 3: Risk of bias assessment	6](https://docs.google.com/document/d/17LwDdoGhF9zScsCQCgruj1KYc0ySs53nuXKCO7MPmNA/edit?tab=t.0#heading=h.fan4dhzhkb3)

Supplementary data 4: Forest plot of anti-IFNα seroprevalence in patients with severe SARS-CoV-2 infection 15

Supplementary data 5: Forest plot of anti-IFNα seroprevalence in uninfected healthy individuals 16

Supplementary data 6: Forest plot of anti-IFNα seroprevalence in hospitalised patients vs patients in the community with SARS-CoV-2 infection 17

[Supplementary data 7. Methodology for measurements of anti-interferon auto-antibody binding for all studies included in meta-analysis	18](https://docs.google.com/document/d/17LwDdoGhF9zScsCQCgruj1KYc0ySs53nuXKCO7MPmNA/edit?tab=t.0#heading=h.557qm7nhpkjw)

[Supplementary data 8. Methodology for measurement of in vitro interferon neutralisation for all studies included in meta-analysis	25](https://docs.google.com/document/d/17LwDdoGhF9zScsCQCgruj1KYc0ySs53nuXKCO7MPmNA/edit?tab=t.0#heading=h.7bs1uccjqcs8)

[Supplementary data 9. Summary of case reports and case series of anti-IFN auto-Ab detected in infectious diseases	29](https://docs.google.com/document/d/17LwDdoGhF9zScsCQCgruj1KYc0ySs53nuXKCO7MPmNA/edit?tab=t.0#heading=h.lebljrco4d64)

[Supplementary data 10. Summary of prevalence of auto-Abs against IFNγ (type 2 IFN) and IFNλ (type 3 IFN) in patients with SARS-CoV-infection of any severity	23](https://docs.google.com/document/d/17LwDdoGhF9zScsCQCgruj1KYc0ySs53nuXKCO7MPmNA/edit?tab=t.0#heading=h.kywucp890v75)

**Supplementary data 1: Search strategy**

Search strategy:

1        	exp Interferons/

2        	(interferon* or interferon-alpha or interferon-beta or interferon-lambda or interferon-gamma or interferon-kappa or interferon-epsilon or interferon-omega or type-I-interferon* or type-II-interferon* or type-III-interferon* or IFN*).mp. [mp=title, book title, abstract, original title, name of substance word, subject heading word, floating sub-heading word, keyword heading word, organism supplementary concept word, protocol supplementary concept word, rare disease supplementary concept word, unique identifier, synonyms, population supplementary concept word, anatomy supplementary concept word]

3        	Autoantibodies/

4        	(autoantibod* or auto-antibod* or "auto antibod*" or "neutrali* antibod*" or "antibod* against interferon*" or (antibod* adj5 interferon*) or (autoantibod* adj5 interferon*)).mp. [mp=title, book title, abstract, original title, name of substance word, subject heading word, floating sub-heading word, keyword heading word, organism supplementary concept word, protocol supplementary concept word, rare disease supplementary concept word, unique identifier, synonyms, population supplementary concept word, anatomy supplementary concept word]

5        	exp Infections/

6        	infectio*.mp. [mp=title, book title, abstract, original title, name of substance word, subject heading word, floating sub-heading word, keyword heading word, organism supplementary concept word, protocol supplementary concept word, rare disease supplementary concept word, unique identifier, synonyms, population supplementary concept word, anatomy supplementary concept word]

7        	1 or 2

8        	3 or 4

9        	5 or 6

10      	7 and 8 and 9

**Supplementary data 2: Prospero form**

Review title

Seroprevalence of anti-interferon auto-antibodies and associated mortality in individuals with infectious diseases

Review objectives

1. What is the seroprevalence of anti-interferon (IFN) auto-antibodies in individuals with infectious diseases?
2. What is the seroprevalence of neutralising anti-interferon (IFN) auto-antibodies in individuals with infectious diseases?
3. What is the seroprevalence of anti-IFN auto-antibodies in individuals hospitalised with infectious diseases?
4. In individuals with infectious diseases does anti-IFN auto-antibodies seropositivity increase risk of 28 day mortality?

SEARCHING AND SCREENING

Searches

We will search three electronic peer-review literature databases (MEDLINE, EMBASE, Cochrane Database of Systematic Reviews) from January 1, 1980 until 1st June 2023 using a comprehensive set of search terms developed in consultation with a university health sciences librarian. We will recheck for relevant studies up to the point of submission. We shall search bibliographies of relevant articles. We will only include English language studies. We will include pre-print literature. We will include case studies, observational studies of any design and any trial studies.

Study design

No restrictions on the types of human studies

ELIGIBILITY CRITERIA

Condition or domain being studied

The disease being studied would be any infectious disease including bacterial, viral, fungal, parasitic and other pathogens. We expect the most abundant literature to be found for acute respiratory virus infections, particularly SARS-CoV-2 and influenza.

Population

Inclusion: Adults and children with any infectious disease (a disease with a known microorganism origin).
Exclusion: Animal studies. Studies including only individuals with known phenotypic autoimmune diseases with high prevalence of auto-antibodies such as SLE. Studies reporting only vaccination outcomes for infectious diseases.

Intervention(s) or exposure(s)

We will include studies including participants with a diagnosis of an infectious disease and any measurements of auto-antibodies using any technique against any interferon subtype at any time in relation to the infection episode. Infectious diseases are defined as diseases with a known microorganism origin including those which are communicable diseases or zoonotic infections.

Comparator(s) or control(s)

Outcome of 28-day mortality will be compared between auto-antibody positive and auto-antibody negative groups of patients with infectious diseases.

Context

OUTCOMES TO BE ANALYSED

Main outcomes

Seroprevalence of anti-interferon auto-antibodies in infectious diseases.
Seroprevalence of neutralising anti-interferon auto-antibodies in infectious diseases. Where this function of auto-antibody is reported.

Additional outcomes

Seroprevalence of anti-interferon auto-antibodies in acute and chronic infectious diseases. Chronic is defined as more than 6 months in duration.
 28-day mortality in individuals with acute infection

DATA COLLECTION PROCESS

Data extraction (selection and coding)

Study selection:
- Number of reviewers applying eligibility criteria and selecting studies for inclusion in the systematic review: two
- How this will be done: independent screening of records with a third author decider in cases of disagreement
- Researchers will be blind to each others decisions
Data extraction:
 - Included articles will be uploaded into a database.
- Data which will be extracted from study documents: study design and methodology, study population characteristics (age, sex, ethnicity, hospitalisation status at time of study), specific infectious disease, class of infectious disease (virus, bacteria, fungus, parasite, other), chronicity of infectious disease (acute <6 months; chronic >6 months), seroprevalence of auto-antibodies (all IFN proteins, type I IFN, type II IFN, type III IFN), prevalence of neutralising auto-antibodies, 28-day all-cause mortality
 - Number of people extracting or checking received data: two
- How this will be done: one person independently extracting data with a second person checking the data
- How disagreements between individuals will be resolved: third party

Risk of bias (quality) assessment

Joanna Briggs Critical Appraisal Checklist for Prevalence Studies (JBI). Two reviewers will produce two independent assessments for risk of bias in selected studies for items 1 and 5 of JBI. The other seven items will be automatically completed based on the other data elements extracted for the review. The nine items will be synthesised to generate a risk of bias assessment (low, moderate, high, unclear) determined using a decision rule (agreed by all authors).

PLANNED DATA SYNTHESIS

Strategy for data synthesis

Criteria under which the data will be synthesised:
The purpose of the analysis is to gather, synthesize, critically appraise, and present the best available estimates of seroprevalence in the relevant population to inform future studies of this emerging biology.
Which data will be synthesised including outcomes and summary effect measures:
We will summarize findings from all included studies. We will synthesize prevalence estimates with 95% confidence intervals for which data is available (stratified by age (over 75, under 75 years), sex, specific infectious disease, class of infectious disease, chronicity of infectious disease). Depending on available data we will aggregate studies with random-effects meta-analysis where deemed appropriate.
The formal method of combining individual study data: Manual data extraction.

Analysis of subgroups or subsets

Subgroups will include: specific infectious disease (by organism), class of infectious disease (virus, bacteria, fungus, parasite, other), chronicity of infectious disease (acute <6 months; chronic >6 months).
Sensitivity analysis will also be stratified by risk of bias (high, medium, low, unclear).

REVIEW AFFILIATION, FUNDING AND PEER REVIEW

Review team members

- Dr Robert Hill, Division of Infection and Immunity, University College London
- Dr Douglas Fink, Division of Infection and Immunity, University College London

Review affiliation

Division of Infection and Immunity, University College London

Funding source

None

Named contact

Dr Douglas Fink, Institute of Immunity and Transplantation, Pears Building, London, NW3 2PP; d.fink@ucl.ac.uk

TIMELINE OF THE REVIEW

Review timeline

Start date: 01 June 2023. End date: 01 November 2023

Date of first submission to PROSPERO

24 July 2023

Date of registration in PROSPERO

25 July 2023

**Supplementary data 3: Risk of bias assessment**

| Author | Sample frame | Study participants recruited appropriately? | Sample size | Study subjects and setting described in detail | Data anaylsis suficient coverage | Valid methods for identification of condition | Condition measured in a standard reliable way | Appropriate statistical analysis | Response rate adequare | Overall appraisal | Risk of bias |
| --- | --- | --- | --- | --- | --- | --- | --- | --- | --- | --- | --- |
| gervais 2024 [1] | yes | yes | yes | yes | yes | yes | yes | yes | yes | Yes | Low |
| gervais 2024 [2] | yes | yes | yes | yes | yes | yes | yes | yes | yes | Yes | Low |
| lin 2023 [3] | yes | yes | yes | yes | yes | yes | yes | yes | yes | Yes | Low |
| gervais 2023 [4] | yes | yes | yes | yes | yes | yes | yes | yes | yes | Yes | Low |
| cockx 2024 [5] | yes | yes | yes | yes | yes | yes | yes | yes | yes | Yes | Low |
| arrestier 2022 [6] | yes | yes | yes | yes | yes | yes | yes | yes | yes | Yes | Low |
| aoki 2024 [7] | yes | yes | yes | yes | yes | yes | yes | yes | yes | Yes | Low |
| pons 2023 [8] | yes | yes | yes | yes | yes | yes | yes | yes | yes | Yes | Low |
| sharif-askari 2023 [9] | yes | yes | yes | yes | yes | yes | yes | yes | yes | Yes | Low |
| sharif-askari 2024 [10] | yes | yes | yes | yes | yes | yes | yes | yes | yes | Yes | Low |
| shi 2024 [11] | yes | yes | yes | yes | yes | yes | yes | yes | yes | Yes | Low |
| joly 2023 [12] | yes | no | yes | yes | yes | yes | yes | yes | yes | Yes | Low |
| farias 2023 [13] | yes | yes | yes | yes | yes | yes | yes | yes | yes | Yes | Low |
| jonassen 2024 [14] | yes | yes | yes | yes | yes | yes | yes | yes | yes | Yes | Low |
| le stang 2024 [15] | yes | yes | yes | yes | yes | yes | no | yes | yes | Yes | Low |
| tiniakou 2024 [16] | yes | yes | yes | yes | yes | yes | yes | yes | yes | Yes | Low |
| von stemann 2024 [17] | yes | yes | yes | yes | yes | yes | yes | yes | yes | Yes | Low |
| Vanker 2023 [18] | yes | yes | yes | yes | yes | yes | yes | yes | yes | Yes | Low |
| Philippot 2023 [19] | yes | yes | yes | yes | yes | yes | yes | yes | yes | Yes | Low |
| Lee 2023 [20] | yes | yes | no | yes | yes | yes | yes | yes | yes | Yes | Low |
| Lebedin 2023 [21] | yes | no | yes | no | yes | no | no | yes | yes | No | High |
| Jiang 2023 [22] | no | yes | no | no | no | yes | no | yes | yes | No | High |
| Feng 2023 [23] | no | no | no | yes | no | no | no | no | yes | No | High |
| Chen 2023 [24] | yes | yes | yes | yes | yes | yes | yes | yes | NA | Yes | Low |
| Cennamo 2023 [25] | no | no | yes | yes | yes | no | no | yes | yes | No | Moderate |
| Alotaibi 2023 [26] | yes | yes | yes | yes | yes | yes | yes | yes | yes | Yes | Low |
| Zhang 2022 [27] | yes | yes | yes | yes | yes | yes | yes | yes | yes | Yes | Low |
| Troya 2022 [28] | no | yes | yes | yes | yes | yes | yes | yes | yes | Yes | Low |
| Steels 2022 [29] | yes | yes | yes | yes | yes | yes | yes | yes | yes | Yes | Low |
| Soltani-Zangbar 2022 [30] | yes | yes | yes | yes | yes | yes | yes | yes | yes | Yes | Low |
| Smith 2022 [31] | yes | yes | yes | yes | yes | yes | yes | yes | yes | Yes | Low |
| Simula 2022 [32] | no | no | no | no | no | no | no | no | no | No | High |
| Shaw 2022 [33] | yes | no | no | yes | no | no | no | yes | no | No | High |
| Scordio 2022 [34] | no | no | yes | yes | yes | yes | yes | yes | yes | Yes | Low |
| Raadsen 2022 [35] | yes | yes | yes | yes | yes | yes | yes | yes | yes | Yes | Low |
| Qiu 2022 [36] | no | no | no | no | no | no | no | no | no | No | High |
| Manry 2022 [37] | yes | no | yes | no | no | no | no | no | yes | No | High |
| Lamacchia 2022 [38] | no | yes | yes | yes | yes | yes | yes | yes | yes | Yes | Low |
| Grimm 2022 [39] | no | yes | yes | yes | no | no | no | no | no | No | High |
| Ghale 2022 [40] | yes | yes | no | yes | yes | yes | yes | yes | yes | Yes | Low |
| Frasca 2022 [41] | yes | yes | yes | yes | yes | yes | yes | yes | yes | Yes | Low |
| Credle 2022 [42] | yes | yes | yes | yes | no | no | no | no | yes | No | Moderate |
| Chen 2022 [43] | no | no | no | no | no | no | no | no | no | No | High |
| Chen 2022 [44] | no | no | no | no | no | no | no | no | no | No | High |
| Chauvineau-Grenier 2022 [45] | yes | yes | yes | yes | yes | yes | yes | yes | yes | Yes | Low |
| Busnadiego 2022 [46] | yes | yes | yes | yes | yes | yes | yes | yes | yes | Yes | Low |
| Bastard 2022 [47] | yes | yes | yes | yes | yes | yes | yes | yes | yes | Yes | Low |
| Barhoum 2022 [48] | no | no | yes | yes | yes | yes | yes | yes | yes | Yes | Low |
| Akbil 2022 [49] | yes | yes | yes | yes | yes | yes | yes | yes | yes | Yes | Low |
| Vazquez 2021 [50] | no | no | no | no | no | no | no | no | no | No | High |
| van der Wijst 2021 [51] | yes | no | yes | yes | yes | yes | yes | yes | yes | Yes | Low |
| Solanich 2021 [52] | yes | yes | yes | yes | yes | yes | yes | yes | yes | Yes | Low |
| Lopez 2021 [53] | yes | yes | yes | yes | yes | yes | yes | yes | yes | Yes | Low |
| Koning 2021 [54] | yes | yes | yes | no | no | no | no | no | yes | No | High |
| Goncalves 2021 [55] | yes | yes | yes | yes | yes | yes | yes | yes | yes | Yes | Low |
| Chen 2021 [56] | yes | yes | yes | yes | yes | yes | yes | yes | yes | Yes | Low |
| Bastard 2021 [57] | yes | yes | yes | yes | yes | yes | yes | yes | yes | Yes | Low |
| Acosta-Ampudia 2021 [58] | yes | yes | yes | yes | no | yes | no | no | no | No | Moderate |
| Abers 2021 [59] | yes | yes | yes | yes | yes | yes | yes | yes | yes | Yes | Low |
| Yoshizawa 2020[60] | yes | yes | yes | no | no | no | no | no | no | No | High |
| Wu 2020 [61] | no | yes | yes | yes | yes | yes | yes | yes | yes | Yes | Low |
| Hong 2020 [62] | no | no | no | no | no | no | no | no | no | No | High |
| Guo 2020 [63] | yes | yes | yes | yes | yes | yes | yes | yes | yes | Yes | Low |
| Bastard 2020 [64] | yes | yes | yes | yes | yes | yes | yes | yes | yes | Yes | Low |
| Zeng 2019 [65] | no | no | no | no | no | no | no | no | no | No | High |
| Jutivorakool 2019 [66] | no | no | no | no | no | no | no | no | no | No | High |
| Hong 2019 [62] | no | no | no | no | no | no | no | no | no | No | High |
| Angkasekwinai 2019 [67] | no | no | no | no | no | no | no | no | no | No | High |
| Wipasa 2018 [68] | no | no | no | no | no | no | no | no | no | No | High |
| Kham-Ngam 2018 [69] | no | no | no | no | no | no | no | no | no | No | High |
| Jutivorakool 2018 [70] | no | no | no | no | no | no | no | no | no | No | High |
| Chetchotisakd 2018 [71] | no | no | no | no | no | no | no | no | no | No | High |
| Aoki 2018 [72] | yes | yes | yes | yes | yes | yes | yes | yes | yes | Yes | Low |
| Suarez 2017 [73] | no | no | no | no | no | no | no | no | no | No | High |
| Phoompoung 2017 [74] | no | no | no | no | no | no | no | no | no | No | High |
| Chetchotisakd 2017 [75] | yes | yes | yes | yes | yes | yes | yes | yes | yes | Yes | Low |
| Kobayashi 2016 [76] | no | no | no | no | no | no | no | no | no | No | High |
| Chi 2016 [77] | yes | no | yes | yes | yes | yes | yes | yes | yes | Yes | Low |
| Bayat 2015 [78] | no | no | yes | no | no | no | no | no | yes | No | High |
| Wipasa 2014 [79] | yes | yes | yes | no | no | no | no | no | no | No | High |
| Kim 2014 [80] | no | no | no | no | no | no | no | no | no | No | High |
| Wongkulab 2013 [81] | no | no | no | no | yes | yes | yes | yes | yes | No | Moderate |
| Browne 2012 [82] | yes | yes | yes | yes | yes | yes | yes | yes | yes | Yes | Low |
| Patel 2005 [83] | yes | yes | yes | no | no | no | no | no | no | No | High |
| Elkarim 1999 [84] | no | no | no | no | no | no | no | no | no | No | High |
| Madariaga 1998 [85] | no | no | no | no | no | no | no | no | no | No | High |
| Bonfanti 1995 [86] | no | no | no | no | no | no | no | no | no | No | High |
| Turano 1992 [87] | no | no | no | no | no | no | no | no | no | No | High |
| Ikeda 1991 [88] | yes | yes | yes | no | no | no | no | no | no | No | High |
| von Wussow 1990 [89] | no | no | no | no | no | no | no | no | no | No | High |
| Caruso 1990 [90] | no | no | no | no | no | no | no | no | no | No | High |
| Eto 2022 [91] | no | yes | yes | no | no | no | yes | yes | yes | No | High |
| Troya 2021 [92] | no | no | no | yes | yes | no | no | yes | yes | No | High |
| Savvateeva 2021 [93] | yes | yes | yes | yes | yes | yes | yes | yes | yes | Yes | Low |
| Zeigler 2021 [93] | yes | yes | yes | no | no | no | no | no | no | No | High |
| Carapito 2021 [94] | yes | yes | yes | yes | yes | yes | yes | yes | yes | Yes | Low |
| Peel 2024 [95] | no | no | no | no | no | no | no | no | no | No | High |

**Supplementary data 4: Forest plot of anti-IFNα seroprevalence in patients with severe SARS-CoV-2 infection**

Group A illustrates studies using auto-ab detection by auto-ab binding, and group B detection by IFN neutralisation assays. 95% prediction intervals shown.

**Supplementary data 5: Forest plot of anti-IFNα seroprevalence in uninfected healthy individuals**

Group A illustrates studies using auto-ab detection by auto-ab binding, and group B detection by IFN neutralisation assays. 95% prediction intervals shown.

**Supplementary data 6: Forest plot of anti-IFNα seroprevalence in hospitalised patients vs patients in the community with SARS-CoV-2 infection**

Group A illustrates patients hospitalised with SARS-CoV-2, and group B patients in the community with SARS-CoV-2. 95% prediction intervals shown.

**Supplementary data 7: Methodology for measurements of anti-interferon auto-antibody binding for all studies included in meta-analysis**

* = Healthy control demographics and/or clinical characteristics not clearly described

| Study | Method | Cut-off methodology reported | Control population |
| --- | --- | --- | --- |
| Vanker, 2023 [18] | Luciferase based immunoprecipitation system | A luminescence signal 30 times higher than the mean value of the healthy controls ran with each LIPS assay | 3 serum samples of known autoantibody negative control serum |
| Philippot, 2023 [19] | Gyros | >100 Gyros units. Methodology as used originally in Bastard 2021 | Not stated |
| Chen, 2023 [24] | ELISA | A “positive” ELISA result was defined as an anti-IFN-γ autoAbs titer ≥30 U/ml, and the cut-off value was defined as the mean value of HC subjects plus 3-fold standard deviationA “positive” ELISA result was defined as an anti-IFN-γ autoAbs titer ≥30 U/ml, and the cut-off value was defined as the mean value of HC subjects plus 3-fold standard deviation | Twenty-two healthy controls without autoimmune diseases or previous SARS-CoV-2 infection |
| Troya, 2022 [28] | Not stated | Not stated | Not stated |
| Steels, 2022 [29] | STAT1 phosphorylation | IFN-alpha2 dose 10ng/ml | Healthy non-infected individuals* |
| Soltani-Zangbar, 2022 [30] | ELISA | The calculated cut-off index was 0.748 nm | Age-matched 50 healthy staff of the corona ward with negative history of Covid-19 infection |
| Smith, 2022 [31] | Gyros | > 95 Gyros units. Methodology as used originally in Bastard, 2021. | Uninfected male healthy controls of ages 31, 53, 81 and 85 |
| Scordio, 2022 [34] | Bioassay based on IFN-  induced inhibition of encephalomyocarditis virus (EMCV) cytopathic effect (CPE) on human lung  carcinoma epithelial cells (A549) | 10 tenfold reduction unit (TRU)/ ml where one TRU was the serum dilution able to reduce IFN titer from 10 to 1 IU/ml | Healthy control* |
| Arrestier, 2022 [6] | Luciferase reporter assay | Samples were considered neutralizing if luciferase induction, normalized against *Renilla* luciferase activity, was below 15% of the median values for controls tested the same day | Healthy control* |
| Lamacchia, 2022 [38] | ELISA | Not stated | Young (< 40 years), sex-matched, healthy unvaccinated patients |
| Frasca, 2022 [41] | ELISA | Not stated | Nineteen gender and age matched healthy controls |
| Chauvineau-Grenier, 2022 [45] | Gyros | >100 Gyros units. Methodology as used originally in Bastard 2021 | Not stated |
| Chauvineau-Grenier, 2022 [45] | ELISA | Not stated | Not stated |
| Busnadiego, 2022 [46] | Bead based assay | MFI values above 10 SDs for IFNα2, or 5 SDs for IFNω were considered positive | Pre-pandemic healthy adults |
| Bastard, 2022 [47] | Gyros | Not stated | Patients with asymptomatic or mild SARS-CoV-2 |
| Bastard, 2022 [47] | Radioligand binding assay | A positive signal was defined as greater than 6 standards deviations above the mean of pre–COVID-19 blood bank non-inflammatory controls | Pre-Covid-19 blood bank non-inflammatory controls |
| Barhoum, 2022 [48] | ELISA | The positivity threshold was 15 ng/mL | Healthy SARS-CoV-2 negative individuals |
| Akbil, 2022 [49] | Reverse ELISA | Samples were considered positive when the respective LSC value exceeded the 97.5th percentile of AAB levels of the analyzed sera from the healthy control | Healthy controls recruited from a study on SARS-CoV2 exposition in health care workers |
| Van der Wijst, 2021 [51] | Radioligand Binding Assay | For the COVID-19 patient and CCP cohorts, a positive signal was defined as greater than 6 SDs above the mean of pre–COVID-19 blood bank noninflammatory controls | Healthy pre-Covid-19 control plasma from the New York Blood Centre within the age range of the patients with Covid-19. |
| Solanich, 2021 [52] | ELISA | Considered as positive results of both auto-Abs against type I IFNs any result greater than a cutoff value calculated as the mean value plus two standard deviations of a control group of healthy non-COVID-19 patients with a similar age and gender | Healthy non-Covid-19 patients with a similar age and gender |
| Lopez, 2021 [53] | ELISA | Not stated | Uninfected healthy control* |
| Bastard, 2021 [57] | ELISA | Optical density > 0.5 | Healthy control* |
| Bastard, 2021 [57] | Gyros | >100 Gyros units | Healthy control* |
| Abers, 2021 [59] | Not clear but methodology references Bastard, 2020 | Not clear but methodology references Bastard, 2020 | Not clear but methodology references Bastard, 2020 |
| Bastard 2020 [64] | Multiplex particle based assay | Patients with an FI of >1500 for IFN-α2 or IFN-β or >1000 for IFN-ω were tested for blocking activity, as were patients positive for another cytokine | Healthy controls* whose samples were collected before the Covid-19 pandemic |
| Bastard 2020 [64] | ELISA | Not stated | Healthy controls* whose samples were collected before the Covid-19 pandemic |
| Bastard, 2020 [64] | Luciferase immunoprecipitation system | The results are expressed in arbitrary units (AU), as a fold-difference relative to the mean of the negative control samples | Healthy controls* whose samples were collected before the Covid-19 pandemic |
| Raadsen, 2022 [35] | ELISA | The 99th percentile IFN-α2 Abs concentration found in healthy controls was 165 ng/mL, which was used as the cutoff to define IFN-α2 Abs positive sera | Pre-pandemic healthy controls* enrolled in a study at the travel medicine and vaccination clinic of Erasmus MC |
| Aoki, 2024 [7] | ELISA | Not clear but methodology references Aoki, 2018 | Healthy controls* |
| Pons, 2023 [8] | ELISA | 1.4 ng/mL as the detection limit and a mean of 17.4 ng/mL as the positivity cutoff for anti-IFN-α auto-Abs in a cohort of healthy controls | Healthy controls* |
| Sharif-Askari, 2023 [9] | ELISA | Not stated but methodology references Bastard, 2021 | Healthy controls* |
| Sharif-Askari, 2024 [10] | ELISA | The cutoff value for ELISA was set at 0.5 optical density (OD) | PBS |
| Farias, 2023 [13] | ELISA | Not stated | Rabbit polyclonal antibody against human IFN-α (Vinci-Biochem) was included as a positive control |
| Jonassen, 2024 [14] | ELISA | A cut-off of 0.5 (blank-corrected OD450-OD630) was used to determine positivity in plasma samples | Healthy control*. Methodology references Fahnoe, 2022 for details |
| Tiniakou, 2024 [16] | ELISA | The cut‐off (calibrated OD units) for assigning antibody positivity, as the mean + 3SD of healthy controls, was 0.075 (anti‐IFNα ELISA) | Healthy controls* |
| Von Stemann, 2024 [17] | Particle based assay | Patients were considered to have high titers of c-aAb if their mean fluorescence intensities (MFIs) were above the 90th percentiles at baseline or the earliest available sample for the seven individual c-aAb. | Not stated |
| Savvateeva, 2021 [93] | Microarray | The cutoff values for selecting positive signals were In/Iref ≥ 2.2 for IgG antibodies and In/Iref ≥ 3.8 for IgM antibodies. | Pre-pandemic healthy controls* |
| Goncalves, 2021 [55] | ELISA | The positive value for anti-IFN-α2 antibody detection was 34ng/mL. The method for detection of autoantibodies for other IFNs was as described in Bastard, 2020. | Pre-pandemic healthy controls* recruited among donors to the Lyon blood transfusion centre |
| Carapito, 2021 [94] | ELISA | Not stated | Uninfected sex and age matched healthy controls |

**Supplementary data 8. Methodology for measurement of *in vitro* interferon neutralisation for all studies included in meta-analysis**

* = Healthy control demographics and/or clinical characteristics not clearly described

| Study | Method | Cut-off methodology reported | Control population |
| --- | --- | --- | --- |
| Vanker, 2023 [18] | Luciferase reporter system | Samples were considered neutralizing if luciferase induction, normalized against *Renilla* luciferase activity, was below 15% of the median values for controls tested the same day. 100pg/ml | Non-neutralising healthy control* samples |
| Vanker, 2023 [18] | Cell-based neutralisation assay | Samples were considered neutralizing if optical density values were reduced below 15% of the mean values for non-neutralizing control sera tested the same day. | Non-neutralising healthy control* samples |
| Philippot, 2023 [19] | Luciferase reporter system | RLA ratio of no more than 3. 100pg/ml | Not stated |
| Chen, 2023 [24] | STAT1 phosphorylation | IFN-gamma dose 20ng/ml. | Twenty-two healthy controls without autoimmune diseases or previous SARS-CoV-2 infection |
| Troya, 2022 [28] | Bastard, 2021 | Bastard, 2021 | Bastard, 2021 |
| Steels, 2022 [29] | STAT1 phosphorylation | IFN-alpha2 dose 10ng/ml | Healthy non-infected individuals |
| Arrestier, 2022 [6] | Luciferase reporter assay | Samples were considered neutralizing if luciferase induction, normalized against *Renilla* luciferase activity, was below 15% of the median values for controls tested the same day | Healthy control |
| Frasca, 2022 [41] | Bioassay | 10 tenfold reduction unit (TRU)/ ml where one TRU was the serum dilution able to reduce IFN titer from 10 to 1 IU/ml | Controls included a titration of each IFN-I preparation |
| Chauvineau-Grenier, 2022 [45] | Luciferase reporter system | Samples were considered neutralizing if luciferase induction, normalized against *Renilla* luciferase activity, was below 15% of the median values for controls tested the same day | Healthy control |
| Busnadiego, 2022 [46] | Luciferase reporter system | Not stated | Pre-pandemic healthy controls* |
| Bastard, 2022 [47] | Luciferase reporter assay | Samples were considered neutralizing if luciferase induction, normalized against *Renilla* luciferase activity, was below 15% of the median values for controls tested the same day | Healthy vaccinated controls 2 - 8 weeks after the mRNA vaccine* |
| Akbil, 2022 [49] | Virus infection based assay | IFN-AABs in a given serum scored specific when preincubation with the highest concentration of IFN-α2 or IFN-⍵ resulted in an at least four-fold reduction of LSC in comparison to analysis of the identical serum without IFN-α2 or IFN-⍵ pre-incubation | Healthy controls recruited from a study on SARS-CoV2 exposition in health care workers |
| Van der Wijst, 2021 [51] | Luciferase reporter assay | RLBA value > 6 standard deviations above healthy controls. | Healthy control* |
| Solanich, 2021 [52] | Luciferase reporter assay | Not stated but methodology references Bastard, 2020 | Healthy non-Covid-19 patients with a similar age and gender |
| Bastard, 2021 [57] | Luciferase reporter assay | Samples were considered neutralizing if luciferase induction, normalized against *Renilla* luciferase activity, was below 15% of the median values for controls tested the same day | Healthy controls* |
| Abers, 2021 [59] | Luciferase reporter assay | Not stated | Not stated |
| Bastard, 2020 [64] | STAT1 phosphorylation | Complete abolition of STAT1 phosphorylation | Pre-pandemic healthy control* |
| Raadsen, 2022 [35] | Virus infection based assay | Sera were considered neutralizing if they reduced IFN-α2 activity by at least 50% | Pre-pandemic healthy controls* enrolled in a study at the travel medicine and vaccination clinic of Erasmus MC |
| Aoki, 2024 [7] | STAT1 phosphorylation | Not clear but methodology references Aoki, 2018 | Healthy controls* |
| Sharif-Askari, 2024 [10] | Luciferase reporter assay | Plasma samples with an induction value of <0.15 were considered positive for neutralizing autoantibodies against IFN-I. | Healthy controls* |
| Shi, 2024 [11] | Luciferase reporter assay | The samples were deemed to possess neutralizing activity if luciferase induction, when normalized to Renilla luciferase activity, was less than 15% of the mean control value. | Pre-pandemic healthy controls below the age of 60 |
| Joly, 2023 [12] | Luciferase reporter assay | Samples were considered neutralizing if luciferase induction, normalized against *Renilla* luciferase activity, was below 15% of the median values for controls tested the same day. | Healthy control* blood donors |
| Le Stang, 2024 [15] | Luciferase reporter assay | Samples were considered neutralizing if luciferase induction, normalized against *Renilla* luciferase activity, was below 15% of the median values for controls tested the same day used originally in Bastard 2021 | Not stated |
| Goncalves, 2021 [55] | Luciferase reporter assay | Samples were considered neutralizing if luciferase induction, normalized against *Renilla* luciferase activity, was below 15% of the median values for controls tested the same day used originally in Bastard 2021 | Pre-pandemic healthy controls* |

**Supplementary data 9. Summary of prevalence of auto-Abs against IFNγ (type 2 IFN) and IFNλ (type 3 IFN) in patients with SARS-CoV-infection of any severity**

| Study | Number of participants | Anti-IFN auto-Abs detected | Prevalence of auto-Ab | Method |
| --- | --- | --- | --- | --- |
| Chen, 2023 [24] | 127 | IFNγ | 7.87% | ELISA |
| Vanker, 2023 [18] | 1002 | IFNλ | 3.39% | Luciferase based immunoprecipitation system. |
| Von stemann, 2024 [17] | 97 | IFNγ | 13.4% | Particle based assay |

**Supplementary data 10. Summary of case reports and case series of anti-IFN auto-Ab detected in infectious diseases.**

| Study | Infectious disease | Number of participants | Anti-IFN auto-Abs detected |
| --- | --- | --- | --- |
| Jordan, 2025 [96] | Mycobacterium persicum, Mycobacterium abscessus | 1 | Gamma |
| Shih, 2025 [97] | SARS-CoV-2 | 1 | Alpha |
| Hirata, 2024 [98] | Mycobacterium massiliense | 1 | Gamma |
| An, 2024 [99] | Stenotrophomonas maltophilia, Klebsiella pneumoniae, Candida tropicalis, Human herpesvirus, Mycobacteirum avium, Mycobacterium tuberculosis | 1 | Gamma |
| Barzaghi, 2024 [100] | West Nile Virus, SARS-CoV-2 | 1 | Alpha, Omega |
| Chubachi, 2024 [101] | Mycobacterium intracellulare | 1 | Gamma |
| Li, 2024 [102] | Talaromyces marneffei | 1 | Gamma |
| Ning, 2024 [103] | Mycobacterium intracellulare, Talaromyces marneffei, Mycobacterium tuberculosis | 1 | Gamma |
| O'Regan, 2024 [104] | Mycobacterium abscessus | 1 | Gamma |
| Chen, 2023 [105] | Talaraomyces marneffei | 1 | Gamma |
| Towachiraporn, 2023 [106] | Salmonellosis | 1 | Gamma |
| Wu, 2023 [107] | Talaromycosis, Nocardiosis | 1 | Gamma |
| Du, 2023 [108] | Talaromcyes marneffi | 1 | Gamma |
| Zhong, 2023 [109] | Disseminated Mycobacterium avium | 1 | Gamma |
| Butel-Simoes, 2023 [110] | Disseminated tuberculosis, cytomegalovirus viraemia | 1 | Gamma |
| Wang, 2023 [111] | Talaromyces marneffei, Mycobacterium kansaii, Listeria monocytogenes | 1 | Gamma |
| Cheng, 2023 [112] | Legionella pneumophilia, Mycobacterium intracellulare | 1 | Gamma |
| Martinot, 2023 [113] | Herpes simplex virus type 2 | 1 | Alpha, Omega |
| Lee, 2023 [114] | Melioidosis | 1 | Gamma |
| Tang, 2023 [115] | Mycobacerium colombiense | 9 | Gamma |
| Pan, 2023 [116] | Mycobacterium kansasii | 1 | Gamma |
| Matono, 2023 [117] | Mycobacterium colombiense | 1 | Gamma |
| Goto, 2023 [118] | Nontuberculosis mycobacterial infection | 1 | Gamma |
| Chou, 2023 [119] | Mycobacterium kansasii, Mycobacterium smegmatis | 1 | Gamma |
| Xing, 2023 [120] | Listeria monocytogenes | 1 | Gamma |
| Zhan, 2022 [121] | Mixed mycosis | 13 | Gamma |
| Sun, 2022 [122] | Mycobacterium avium complex | 1 | Gamma |
| Qiu, 2022 [123] | Talaromyces marneffei, mcyobacterium tuberculosis | 1 | Gamma |
| Lo, 2022 [124] | Mycobacterium avium complex | 1 | Gamma |
| Lin, 2022 [125] | Mycobacterium chimaera | 1 | Gamma |
| Kajeekul, 2022 [126] | Francisella | 1 | Gamma |
| Hirayama, 2022 [127] | Mycolicibacter arupensis, Mycobacterium avium | 1 | Gamma |
| Hidekawa, 2022 [128] | Mycobacterium kansasii | 1 | Gamma |
| de Castro, 2022 [129] | SARS-CoV-2 | 3 | Gamma |
| Boyle, 2022 [130] | Mycobacterium avium complex | 1 | Gamma |
| Zeng, 2021 [65] | Talaromyces marneffei, Burkholderia cepacia | 1 | Gamma |
| Zeitler, 2021 [131] | Mycobacterium avium complex | 1 | Gamma |
| Yang, 2021 [132] | Mycobacterium intracellulare | 1 | Gamma |
| Uno, 2021[133] | Mycobacterium avium complex | 1 | Gamma |
| Thapa, 2021 [134] | Mycobacterium abscessus | 1 | Gamma |
| Rocco, 2021 [135] | Mycobacterium intracellulare | 1 | Gamma |
| Peng, 2021 [136] | Salmonella spondylodiscitis | 1 | Gamma |
| Ochoa, 2021 [137] | Mycobacterium avium complex | 1 | Gamma |
| Mochizuka, 2021 [138] | Mycobacterium avium complex | 1 | Gamma |
| King, 2021 [139] | Mycobacterium avium complex | 3 | Gamma |
| Jin, 2021 [140] | Talaromyces marneffei, Mycobacterium abscessus | 1 | Gamma |
| Dahl, 2021 [141] | Mycobacterium avium complex, Salmonella kentucky, Herpes Simplex Virus 2 | 1 | Gamma |
| Asako, 2021 [142] | Mycobacterium abscessus | 1 | Gamma |
| Ansari, 2021 [143] | Varicella zoster virus | 1 | Alpha, Gamma |
| Yamaba, 2020 [144] | Mycobacterium avium complex | 1 | Gamma |
| Yaghnam, 2020 [145] | Mycobacterium avium complex | 1 | Gamma |
| Weng, 2020 [146] | Mycobacterium szulgai | 1 | Gamma |
| Rujirachun, 2020 [147] | Cryptococcus, Varicella zoster virus, Mycobacterium abscessus complex | 1 | Gamma |
| Roerden, 2020 [148] | Cytomegalovirus, Salmonella typhimurium, Mycobacterium intracellulare | 1 | Gamma |
| Liang, 2020 [149] | Talaromyces marneffei, Nontuberculosis mycobacterium | 1 | Gamma |
| Liang, 2020 [150] | Salmonella typhi, Mycobacterium sp, Candida sp, Burkholderia cepaca, Talaromyces marneffei | 1 | Gamma |
| Keragala, 2020 [151] | Mycobacterium simiae | 1 | Gamma |
| Yerramilli, 2019 [152] | Mycobacterium abscessus | 1 | Gamma |
| Yeh, 2019 [153] | Mycobacterium avium complex | 1 | Gamma |
| Xu, 2019 [154] | Mycobacterium avium complex | 1 | Gamma |
| Wongkamhla, 2019 [155] | Talaromyces marneffei | 1 | Gamma |
| Wen, 2019 [156] | Mycobacterium intracellulare | 1 | Gamma |
| Tanaka, 2019 [157] | Mycolicibacterium phlei | 1 | Gamma |
| Su, 2019 [158] | Talaromyces marneffei, Mycobacterium avium complex | 1 | Gamma |
| Namkoong, 2019 [159] | Mycobacterium avium complex | 1 | Gamma |
| Liew, 2019 [160] | Mycobacterium abscessus, Mycobacterium avium complex | 2 | Gamma |
| Koizumi, 2019 [161] | Mycobacterium avium complex | 1 | Gamma |
| Kashihara, 2019 [162] | Mycobacterium kansasii | 1 | Gamma |
| Cheng, 2019 [163] | Mycobacterium abscessus | 1 | Gamma |
| Xu, 2018 [164] | Talaromyces marneffei, Mycobacterium abscessus | 1 | Gamma |
| Miyashita, 2018 [165] | Mycobacterium avium complex | 1 | Gamma |
| Hung, 2018 [166] | Mycobacterium kansasii | 1 | Gamma |
| Wu, 2017 [167] | Mycobacterium avium complex | 1 | Gamma |
| Van de Vosse, 2017 [168] | Histoplasma capsulatum | 1 | Gamma |
| Li, 2017 [169] | Mycobacterium avium complex | 1 | Gamma |
| Koizumi, 2017 [170] | Mycobacterium avium complex | 1 | Gamma |
| Furuya, 2017 [171] | Mycobacterium intracellulare | 1 | Gamma |
| Chang, 2017 [172] | Mycobacterium abscessus | 1 | Gamma |
| Asakura, 2017 [173] | Mycobacterium genavense | 1 | Gamma |
| Xie, 2016 [174] | Mycobacterium tuberculosis | 1 | Gamma |
| Tham, 2016 [175] | Mycobacterium kansasii, Mycobacterium avium complex, haemophilium and kansasii | 3 | Gamma |
| Valour, 2016 [176] | Mycobacterium fortuitum | 1 | Gamma |
| Pruetpongpun, 2016 [177] | Talaromyces marneffei, Mycobacterium abscessus | 1 | Gamma |
| Noguchi, 2016 [178] | Mycobacterium avium complex | 1 | Gamma |
| Liu, 2016 [179] | Mycobacterium abscessus | 1 | Gamma |
| Lee, 2016 [180] | Mycobacterium fortuitum | 1 | Gamma |
| Ikeda, 2016 [181] | Mycobacterium avium | 1 | Gamma |
| Otome, 2015 [182] | Mycobacterium haemophilum | 1 | Gamma |
| Nishimura, 2015 [183] | Mycobacterium avium complex | 1 | Gamma |
| Hanitsch, 2015 [184] | Mycobacterium avium complex, Toxoplasma gondii, Salmonella enterica | 1 | Gamma |
| Wang, 2014 [185] | Mycobacterium abscessus | 1 | Gamma |
| Suzuki, 2014 [186] | Mycobacterium fortuitum | 1 | Gamma |
| O'Connell, 2014 [187] | Mycobacterium avium complex | 1 | Gamma |
| DeLeon, 2014 [188] | Mycobacterium avium complex | 1 | Gamma |
| Czaja, 2014 [189] | Mycobacterium abscessus | 1 | Gamma |
| Chan, 2014 [190] | Mycobacteriodes chelonae | 1 | Gamma |
| Yamaguchi, 2013 [191] | Mycobacterium intracellulare | 1 | Gamma |
| Watanabe, 2013 [192] | Mycobacterium avium complex | 1 | Gamma |
| Poulin, 2013 [193] | Mycobacterium colombiense, Cytomegalovirus, Pseudomonas aeruginosa | 1 | Gamma |
| Nei, 2013 [194] | Mycobacterium kansasii | 1 | Gamma |
| Chaisathaphol, 2013 [195] | Mycobacterium avium | 1 | Gamma |
| Tang, 2010 [196] | Mycobacterium chelonae, Mycobacterium kansasii, Penicillium marneffei, Burkholderia pseudomallei, Mycobacterium fortuitum, Mycobacterium intracellulare, Salmonella enterica | 3 | Gamma |
| Koya, 2009 [197] | Mycobacterium avium complex | 1 | Gamma |
| Baerlecken, 2009 [198] | Mycobacterium intracellulare | 1 | Gamma |
| Kampmann, 2005 [199] | Mycobacterium intracellulare, Mycobacterium fortuitum, Aspergillus | 1 | Gamma |
| Hoflich, 2004 [200] | Mycobacterium chelonae, Burkholderia cocovenenans | 1 | Gamma |
| Doffinger, 2004 [201] | Mycobacterium tuberculosis, Mycobacterium chelonae | 1 | Gamma |
| Pozzetto, 1984 [202] | Varicella zoster virus | 1 | Alpha |

1.	Gervais, A.A.-O., et al., *Auto-Abs neutralizing type I IFNs in patients with severe Powassan, Usutu, or Ross River virus disease. LID - 10.1084/jem.20240942 [doi] LID - e20240942.* (1540-9538 (Electronic)).

2.	Gervais, A.A.-O., et al., *Autoantibodies neutralizing type I IFNs underlie severe tick-borne encephalitis in ∼10% of patients. LID - 10.1084/jem.20240637 [doi] LID - e20240637.* (1540-9538 (Electronic)).

3.	Lin, S.-C., et al., *Blockade of interferon signaling decreases gut barrier integrity and promotes severe West Nile virus disease.* Nature Communications, 2023. **14**(1): p. 5973.

4.	Gervais, A., et al., *Autoantibodies neutralizing type I IFNs underlie West Nile virus encephalitis in ∼40% of patients.* Journal of Experimental Medicine, 2023. **220**(9): p. e20230661.

5.	Cockx, M.A.-O., et al., *IFN-α2 Autoantibody Screening and Functional Evaluation in Viral and Bacterial Infections.* (2576-9456 (Print)).

6.	Arrestier, R., et al., *Auto-antibodies against type I IFNs in > 10% of critically ill COVID-19 patients: a prospective multicentre study.* Annals of Intensive Care, 2022. **12**(1): p. 121.

7.	Aoki, A., et al., *Suppression of Type I Interferon Signaling in Myeloid Cells by Autoantibodies in Severe COVID-19 Patients.* (1573-2592 (Electronic)).

8.	Pons, M.A.-O., et al., *High Anti-Interferon-Alpha Autoantibody Levels in Severe/Critical COVID-19 Patients from Peru.* (1557-7465 (Electronic)).

9.	Saheb Sharif-Askari, F., et al., *Increased blood immune regulatory cells in severe COVID-19 with autoantibodies to type I interferons.* Scientific Reports, 2023. **13**(1): p. 17344.

10.	Saheb Sharif-Askari, N., et al., *Salivary autoantibodies to type I IFNs: Mirror plasma levels, predispose to severe COVID-19, and enhance feasibility for clinical screening.* (1527-3288 (Electronic)).

11.	Shi, D., et al., *Prevalence of Neutralizing Autoantibodies Against Type I Interferon in a Multicenter Cohort of Severe or Critical COVID-19 Cases in Shanghai.* (1573-2592 (Electronic)).

12.	Joly, C., et al., *More rapid blood interferon α2 decline in fatal versus surviving COVID-19 patients.* (1664-3224 (Electronic)).

13.	Farias, T.A.-O., et al., *HLA-DPB1*13:01 associates with enhanced, and KIR2DS4*001 with diminished protection from developing severe COVID-19.* (2059-2310 (Electronic)).

14.	Jonassen, T.B., et al., *Alveolar cytokines and interferon autoantibodies in COVID-19 ARDS.* (1664-3224 (Electronic)).

15.	Le Stang, V., et al., *Similar Kinetics of Pulmonary SARS-CoV-2 Load in Intensive Care Unit Patients with COVID-19 Pneumonia with or Without Autoantibodies Neutralizing Type I Interferons.* (1573-2592 (Electronic)).

16.	Tiniakou, E.A.-O.X., et al., *Autoantibodies in hospitalised patients with COVID-19.* (2050-0068 (Print)).

17.	Von Stemann, J.H., et al., *Autoantibodies targeting interferons and GM-CSF are associated with adverse outcome risk, comorbidities, and pathogen in community-acquired pneumonia.* (1664-3224 (Electronic)).

18.	Vanker, M., et al., *Autoantibodies Neutralizing Type III Interferons Are Uncommon in Patients with Severe Coronavirus Disease 2019 Pneumonia.* Journal of Interferon & Cytokine Research, 2023. **43**(9): p. 379-393.

19.	Philippot, Q.A.-O., et al., *Autoantibodies Neutralizing Type I IFNs in the Bronchoalveolar Lavage of at Least 10% of Patients During Life-Threatening COVID-19 Pneumonia.* (1573-2592 (Electronic)).

20.	Lee, W.A.-O., et al., *Distinct Lymphocyte Immunophenotyping and Quantitative Anti-Interferon Gamma Autoantibodies in Taiwanese HIV-Negative Patients with Non-Tuberculous Mycobacterial Infections.* (1573-2592 (Electronic)).

21.	Lebedin, M.A.-O., et al., *Discriminating promiscuous from target-specific autoantibodies in COVID-19.* (1521-4141 (Electronic)).

22.	Jiang, W.A.-O.X., et al., *COVID-19 is associated with bystander polyclonal autoreactive B cell activation as reflected by a broad autoantibody production, but none is linked to disease severity.* (1096-9071 (Electronic)).

23.	Feng, A., et al., *Autoantibodies are highly prevalent in non-SARS-CoV-2 respiratory infections and critical illness. LID - 10.1172/jci.insight.163150 [doi] LID - e163150.* (2379-3708 (Electronic)).

24.	Chen, P.K., et al., *The detectable anti-interferon-γ autoantibodies in COVID-19 patients may be associated with disease severity.* (1743-422X (Electronic)).

25.	Cennamo, M., et al., *Low Interferon-γ Levels in Cord and Peripheral Blood of Pregnant Women Infected with SARS-CoV-2. LID - 10.3390/microorganisms11010223 [doi] LID - 223.* (2076-2607 (Print)).

26.	Alotaibi, F., et al., *Type I interferon autoantibodies in hospitalized patients with Middle East respiratory syndrome and association with outcomes and treatment effect of interferon beta-1b in MIRACLE clinical trial.* (1750-2659 (Electronic)).

27.	Zhang, Q., et al., *Autoantibodies against type I IFNs in patients with critical influenza pneumonia.* J Exp Med, 2022. **219**(11).

28.	Troya, J.A.-O.X., et al., *Low Lymphocytes and IFN-Neutralizing Autoantibodies as Biomarkers of COVID-19 Mortality.* (1573-2592 (Electronic)).

29.	Steels, S., et al., *Transient Increase of Pre-existing Anti-IFN-α2 Antibodies Induced by SARS-CoV-2 Infection.* (1573-2592 (Electronic)).

30.	Soltani-Zangbar, M.S., et al., *A comprehensive evaluation of the immune system response and type-I Interferon signaling pathway in hospitalized COVID-19 patients.* (1478-811X (Electronic)).

31.	Smith, N.A.-O.X., et al., *Defective activation and regulation of type I interferon immunity is associated with increasing COVID-19 severity.* (2041-1723 (Electronic)).

32.	Simula, E.R., et al., *Increased Presence of Antibodies against Type I Interferons and Human Endogenous Retrovirus W in Intensive Care Unit COVID-19 Patients.* (2165-0497 (Electronic)).

33.	Shaw, E.R., et al., *Temporal Dynamics of Anti-Type 1 Interferon Autoantibodies in Patients With Coronavirus Disease 2019.* (1537-6591 (Electronic)).

34.	Scordio, M., et al., *High frequency of neutralizing antibodies to type I Interferon in HIV-1 patients hospitalized for COVID-19.* Clinical Immunology, 2022. **241**: p. 109068.

35.	Raadsen, M.A.-O., et al., *Interferon-α2 Auto-antibodies in Convalescent Plasma Therapy for COVID-19.* (1573-2592 (Electronic)).

36.	Qiu, Y., et al., *Clinical findings and predictive factors for positive anti-interferon-γ autoantibodies in patients suffering from a non-tuberculosis mycobacteria or Talaromyces marneffei infection: a multicenter prospective cohort study.* (2045-2322 (Electronic)).

37.	Manry, J., et al., *The risk of COVID-19 death is much greater and age dependent with type I IFN autoantibodies.* Proceedings of the National Academy of Sciences, 2022. **119**(21): p. e2200413119.

38.	Lamacchia, G., et al., *Clinical and Immunological Features of SARS-CoV-2 Breakthrough Infections in Vaccinated Individuals Requiring Hospitalization.* Journal of Clinical Immunology, 2022. **42**(7): p. 1379-1391.

39.	Grimm, L., et al., *Immune Dysregulation in Acute SARS-CoV-2 Infection.* (2469-2964 (Electronic)).

40.	Ghale, R., et al., *Prevalence of type-1 interferon autoantibodies in adults with non-COVID-19 acute respiratory failure.* (1465-993X (Electronic)).

41.	Frasca, F., et al., *Anti-IFN-α/-ω neutralizing antibodies from COVID-19 patients correlate with downregulation of IFN response and laboratory biomarkers of disease severity.* (1521-4141 (Electronic)).

42.	Credle, J.J., et al., *Unbiased discovery of autoantibodies associated with severe COVID-19 via genome-scale self-assembled DNA-barcoded protein libraries.* (2157-846X (Electronic)).

43.	Chen, Z.M., et al., *Anti-Interferon-γ Autoantibodies Impair T-Lymphocyte Responses in Patients with Talaromyces marneffei Infections.* (1178-6973 (Print)).

44.	Chen, Y.C., et al., *Clinicopathological Manifestations and Immune Phenotypes in Adult-Onset Immunodeficiency with Anti-interferon-γ Autoantibodies.* (1573-2592 (Electronic)).

45.	Chauvineau-Grenier, A.A.-O.X., et al., *Autoantibodies Neutralizing Type I Interferons in 20% of COVID-19 Deaths in a French Hospital.* (1573-2592 (Electronic)).

46.	Busnadiego, I., et al., *Critically ill COVID-19 patients with neutralizing autoantibodies against type I interferons have increased risk of herpesvirus disease.* (1545-7885 (Electronic)).

47.	Bastard, P.A.-O., et al., *Vaccine breakthrough hypoxemic COVID-19 pneumonia in patients with auto-Abs neutralizing type I IFNs.* (2470-9468 (Electronic)).

48.	Barhoum, P., et al., *Phenotypic Heterogeneity of Fulminant COVID-19--Related Myocarditis in Adults.* J Am Coll Cardiol, 2022. **80**(4): p. 299-312.

49.	Akbil, B., et al., *Early and Rapid Identification of COVID-19 Patients with Neutralizing Type I Interferon Auto-antibodies.* (1573-2592 (Electronic)).

50.	Vazquez, S.E., et al., *Neutralizing Autoantibodies to Type I Interferons in COVID-19 Convalescent Donor Plasma.* (1573-2592 (Electronic)).

51.	van der Wijst, M.A.-O., et al., *Type I interferon autoantibodies are associated with systemic immune alterations in patients with COVID-19.* (1946-6242 (Electronic)).

52.	Solanich, X.A.-O., et al., *Pre-existing Autoantibodies Neutralizing High Concentrations of Type I Interferons in Almost 10% of COVID-19 Patients Admitted to Intensive Care in Barcelona.* (1573-2592 (Electronic)).

53.	Lopez, J.A.-O., et al., *Early nasal type I IFN immunity against SARS-CoV-2 is compromised in patients with autoantibodies against type I IFNs. LID - 10.1084/jem.20211211 [doi] LID - e20211211.* (1540-9538 (Electronic)).

54.	Koning, R., et al., *Autoantibodies against type I interferons are associated with multi-organ failure in COVID-19 patients.* (1432-1238 (Electronic)).

55.	Goncalves, D.A.-O., et al., *Antibodies against type I interferon: detection and association with severe clinical outcome in COVID-19 patients.* (2050-0068 (Print)).

56.	Chen, Z.M., et al., *Clinical findings of Talaromyces marneffei infection among patients with anti-interferon-γ immunodeficiency: a prospective cohort study.* (1471-2334 (Electronic)).

57.	Bastard, P., et al., *Autoantibodies neutralizing type I IFNs are present in ~4% of uninfected individuals over 70 years old and account for ~20% of COVID-19 deaths.* Science Immunology. **6**(62): p. eabl4340.

58.	Acosta-Ampudia, Y., et al., *COVID-19 convalescent plasma composition and immunological effects in severe patients.* (1095-9157 (Electronic)).

59.	Abers, M.A.-O., et al., *Neutralizing type-I interferon autoantibodies are associated with delayed viral clearance and intensive care unit admission in patients with COVID-19.* (1440-1711 (Electronic)).

60.	Yoshizawa, K., et al., *Serum Anti-interferon-γ Autoantibody Titer as a Potential Biomarker of Disseminated Non-tuberculous Mycobacterial Infection.* (1573-2592 (Electronic)).

61.	Wu, U.I., et al., *Incorrect diagnoses in patients with neutralizing anti-interferon-gamma-autoantibodies.* (1469-0691 (Electronic)).

62.	Hong, G.H., et al., *Natural History and Evolution of Anti-Interferon-γ Autoantibody-Associated Immunodeficiency Syndrome in Thailand and the United States.* (1537-6591 (Electronic)).

63.	Guo, J., et al., *Anti-IFN-γ autoantibodies underlie disseminated Talaromyces marneffei infections. LID - 10.1084/jem.20190502 [doi] LID - e20190502.* (1540-9538 (Electronic)).

64.	Bastard, P., et al., *Autoantibodies against type I IFNs in patients with life-threatening COVID-19.* Science, 2020. **370**(6515): p. eabd4585.

65.	Zeng, W., et al., *Talaromyces marneffei and Burkholderia cepacia Co-Infection in a HIV-Uninfected Patient with Anti-Interferon-γ Autoantibodies.* (1178-6973 (Print)).

66.	Jutivorakool, K. and J. Vanichanan, *1369. Clinical Manifestations, Treatment, and Outcome of Nontuberculous Mycobacteria (NTM) Infection in Adult-Onset Immunodeficiency Associated with Anti-interferon-gamma Autoantibodies in King Chulalongkorn Memorial Hospital.* (2328-8957 (Electronic)).

67.	Angkasekwinai, N., et al., *Clinical outcome and laboratory markers for predicting disease activity in patients with disseminated opportunistic infections associated with anti-interferon-γ autoantibodies.* (1932-6203 (Electronic)).

68.	Wipasa, J.A.-O., et al., *Characterization of anti-interferon-γ antibodies in HIV-negative immunodeficient patients infected with unusual intracellular microorganisms.* (1535-3699 (Electronic)).

69.	Kham-Ngam, I., et al., *Epidemiology of and risk factors for extrapulmonary nontuberculous mycobacterial infections in Northeast Thailand.* (2167-8359 (Print)).

70.	Jutivorakool, K., et al., *Skin Manifestations in Patients with Adult-onset Immunodeficiency due to Anti-interferon-gamma Autoantibody: A Relationship with Systemic Infections.* (1651-2057 (Electronic)).

71.	Chetchotisakd, P.A.-O., et al., *Intravenous Cyclophosphamide Therapy for Anti-IFN-Gamma Autoantibody-Associated Mycobacterium abscessus Infection.* (2314-7156 (Electronic)).

72.	Aoki, A., et al., *Clinical Significance of Interferon-γ Neutralizing Autoantibodies Against Disseminated Nontuberculous Mycobacterial Disease.* (1537-6591 (Electronic)).

73.	Suárez, I., et al., *Repurposing QuantiFERON for Detection of Neutralizing Interferon-γ Autoantibodies in Patients With Nontuberculous Mycobacterial Infections.* (1537-6591 (Electronic)).

74.	Phoompoung, P., et al., *Factors associated with acquired Anti IFN- γ autoantibody in patients with nontuberculous mycobacterial infection.* (1932-6203 (Electronic)).

75.	Chetchotisakd, P., et al., *Cryptococcosis in Anti-Interferon-Gamma Autoantibody-Positive Patients: a Different Clinical Manifestation from HIV-Infected Patients.* (1884-2836 (Electronic)).

76.	Kobayashi, T., et al., *Nontuberculous Mycobacterial Osteomyelitis in Human Immunodeficiency Virus-Negative Patients: A Case Series.* Japanese Journal of Infectious Diseases, 2016. **69**(2): p. 149-150.

77.	Chi, C.Y., et al., *Clinical manifestations, course, and outcome of patients with neutralizing anti-interferon-γ autoantibodies and disseminated nontuberculous mycobacterial infections.* (1536-5964 (Electronic)).

78.	Bayat, A., et al., *Anti-cytokine autoantibodies in postherpetic neuralgia.* (1479-5876 (Electronic)).

79.	Wipasa, J., et al., *Cellular Immune Responses in HIV-Negative Immunodeficiency with Anti-Interferon-γ Antibodies and Opportunistic Intracellular Microorganisms.* PloS one, 2014. **9**: p. e110276.

80.	Kim, K., et al., *Levels of anti-cytokine antibodies may be elevated in patients with pulmonary disease associated with non-tuberculous mycobacteria.* (1096-0023 (Electronic)).

81.	Wongkulab, P., et al., *Autoantibody to interferon-gamma associated with adult-onset immunodeficiency in non-HIV individuals in Northern Thailand.* (1932-6203 (Electronic)).

82.	Browne, S.K., et al., *Adult-onset immunodeficiency in Thailand and Taiwan.* (1533-4406 (Electronic)).

83.	Patel, S.Y., et al., *Anti-IFN-gamma autoantibodies in disseminated nontuberculous mycobacterial infections.* (0022-1767 (Print)).

84.	ElKarim, R., et al., *Levels of gamma interferon and interleukin-4 are inversely related to the levels of their corresponding autoantibodies in patients with lower respiratory tract infection.* (0019-9567 (Print)).

85.	Madariaga, L., et al., *Detection of anti-interferon-gamma autoantibodies in subjects infected by Mycobacterium tuberculosis.* (1027-3719 (Print)).

86.	Bonfanti, C., et al., *Increased Levels of Antibodies to IFN-γ in Human and Experimental African Trypanosomiasis.* Scandinavian Journal of Immunology, 1995. **41**(1): p. 49-52.

87.	Turano, A. and A. Caruso, *The role of human autoantibodies against gamma-interferon.* (0305-7453 (Print)).

88.	Ikeda, Y., et al., *Naturally occurring anti-interferon-alpha 2a antibodies in patients with acute viral hepatitis.* (0009-9104 (Print)).

89.	von Wussow, P., et al., *The interferon-induced Mx-homologous protein in people with symptomatic HIV-1 infection.* (0269-9370 (Print)).

90.	Caruso, A., et al., *Natural antibodies to IFN-gamma in man and their increase during viral infection.* (0022-1767 (Print)).

91.	Eto, S., et al., *Neutralizing Type I Interferon Autoantibodies in Japanese Patients with Severe COVID-19.* (1573-2592 (Electronic)).

92.	Troya, J.A.-O.X., et al., *Neutralizing Autoantibodies to Type I IFNs in >10% of Patients with Severe COVID-19 Pneumonia Hospitalized in Madrid, Spain.* (1573-2592 (Electronic)).

93.	Savvateeva, E.A.-O., et al., *Microarray-Based Detection of Antibodies against SARS-CoV-2 Proteins, Common Respiratory Viruses and Type I Interferons. LID - 10.3390/v13122553 [doi] LID - 2553.* (1999-4915 (Electronic)).

94.	Carapito, R.A.-O.X., et al., *Identification of driver genes for critical forms of COVID-19 in a deeply phenotyped young patient cohort.* (1946-6242 (Electronic)).

95.	Peel, J.N., et al., *Neutralizing IFN-γ autoantibodies are rare and pathogenic in HLA-DRB1*15:02 or 16:02 individuals. LID - 10.1172/JCI178263 [doi] LID - e178263.* (1558-8238 (Electronic)).

96.	Jordan, V.A.-O. and R. Pickles, *Disseminated nontuberculous mycobacterial infection in the context of interferon-gamma autoantibody syndrome: A case report and review of the literature.* (2062-509X (Print)).

97.	Shih, Y.S., et al., *Life-threatening COVID-19 in a thymoma patient with anti-interferon-α autoantibodies.* (1995-9133 (Electronic)).

98.	Hirata, M., et al., *Mycobacterium abscessus subspecies massiliense infection with anti-interferon-gamma autoantibodies.* Clinical Infection in Practice, 2024. **21**: p. 100333.

99.	An, R.A.-O., et al., *A patient with positive anti-IFN-γ autoantibody and monoclonal gammaglobulinemia masquerading as multiple myeloma: Case report and literature review.* (2050-0904 (Print)).

100.	Barzaghi, F.A.-O., et al., *Severe West Nile Virus and Severe Acute Respiratory Syndrome Coronavirus 2 Infections in a Patient With Thymoma and Anti-Type I Interferon Antibodies.* (1537-6613 (Electronic)).

101.	Chubachi, K., et al., *Disseminated Non-tuberculous Mycobacteriosis with a Skull Lesion Controlled by Resection after Exacerbation during Antimicrobial Chemotherapy in a Patient Positive for Anti-IFN-γ-neutralizing Autoantibodies.* (1349-7235 (Electronic)).

102.	Li, K., et al., *Case report: Systemic multi-organ involvement in an adult-onset immunodeficiency patient infected with Talaromyces marneffei.* (1664-3224 (Electronic)).

103.	Ning, Y., et al., *Multiple intracellular pathogen infections with ocular pathologies associated with adult-onset immunodeficiency due to anti-interferon-γ autoantibodies: a case report.* (1471-2334 (Electronic)).

104.	O'Regan, R.A.-O. and E.G. Muldoon, *Disseminated Mycobacterium abscessus secondary to adult onset immunodeficiency syndrome due to anti-interferon gamma autoantibodies.* (1399-3062 (Electronic)).

105.	Chen, G., et al., *Accurate diagnosis of bronchopulmonary Talaromyces marneffei infection in an anti-IFN-γ autoantibodies positive patient assisted by endobronchial ultrasound-guided TBNA and mNGS: a case report.* (2235-2988 (Electronic)).

106.	Towachiraporn, S., H. Thongwitokomarn, and P. Salee, *Anti-interferon-gamma autoantibody and salmonellosis: Case report and literature review.* (2214-2509 (Print)).

107.	Wu, S.A.-O., et al., *Brain Nocardiosis and Pulmonary Talaromycosis Infection in a Patient with Anti-IFN-γ Autoantibodies: A Case Report.* (1178-6973 (Print)).

108.	Du, R., Y. Feng, and H. Mao, *Case report: Diagnosis of Talaromyces marneffei infection in an HIV-negative patient with septic shock and high-titer anti-interferon gamma autoantibodies by metagenomic next-generation sequencing.* (2235-2988 (Electronic)).

109.	Zhong, M., et al., *Disseminated Mycobacterium avium infection associated with adult-onset immunodeficiency because of anti-interferon-gamma autoantibodies: a case report.* (1365-4632 (Electronic)).

110.	Butel-Simoes, G.I., et al., *Disseminated tuberculosis, CMV viraemia & haemophagocytic-lymphohistiocystosis syndrome in an adult patient with anti- IFNγ autoantibodies - case report and brief review.* (2772-6134 (Electronic)).

111.	Wang, H., et al., *Multiple refractory intracellular pathogen infections in a human immunodeficiency virus-negative patient with anti-interferon-γ autoantibodies: a case report.* (1471-2334 (Electronic)).

112.	Cheng, H.A.-O., et al., *A rare presentation of Legionella pneumophila and Mycobacterium intracellulare co-infection masquerading as metastatic lung cancer in a patient with positive anti-interferon gamma antibody.* (2051-3380 (Print)).

113.	Martinot, M.A.-O., et al., *Severe acute herpes virus type 2 primo-infection and its association with anti-type 1 interferon autoantibodies.* (1435-4373 (Electronic)).

114.	Lee, C.A.-O., et al., *A fatal case of septicemic melioidosis in a patient with autoantibody against interferon-γ: a case report.* (1435-4373 (Electronic)).

115.	Tang, M., et al., *Clinical features of rare disseminated Mycobacterium colombiense infection in nine patients who are HIV-negative in Guangxi, China.* (1878-3511 (Electronic)).

116.	Pan, C., et al., *Severe Disseminated Mycobacterium kansasii Infection due to Autoantibodies Against IFN-ɣ.* (1573-2592 (Electronic)).

117.	Matono, T., et al., *Diagnostic and Therapeutic Challenges in Disseminated Mycobacterium colombiense Infection Caused by Interferon-γ Neutralizing Autoantibodies.* Open Forum Infect Dis, 2023. **10**(2): p. ofad035.

118.	Goto, R., et al., *Disseminated nontuberculous mycobacterial infection in a patient with idiopathic CD4 lymphocytopenia and IFN-γ neutralizing antibodies: a case report.* (1471-2334 (Electronic)).

119.	Chou, Y.T., W.A. Liao, and C.A.-O. Kuo, *Mixed Mycobacterium kansasii and Mycobacterium smegmatis infection in an adult-onset immunodeficiency patient with anti-interferon-γ autoantibodies.* (2051-3380 (Print)).

120.	Xing, F., et al., *Next-Generation Sequencing-Based Diagnosis of Bacteremic Listeria monocytogenes Meningitis in a Patient with Anti-Interferon Gamma: Autoantibodies: A Case Report.* 2641-5917Infectious Microbes & Diseases, 2022. **4**(1).

121.	Zhan, Y., et al., *Successful Management of Mixed Mycosis in HIV-Negative Patients With Different Immune Status: A Case Series Report.* (2235-2988 (Electronic)).

122.	Sun, R.L., et al., *Osteoblastic flare phenomenon in a patient treated for disseminated non-tuberculous mycobacterial infection.* (1468-3296 (Electronic)).

123.	Qiu, Y., et al., *Talaromyces marneffei and Mycobacterium tuberculosis co-infection in a patient with high titer anti-interferon-γ autoantibodies: a case report.* (1471-2334 (Electronic)).

124.	Lo, T.H., et al., *Periocular Infection of Mycobacterium avium Complex in a Patient with Interferon-γ Autoantibodies: A Case Report. LID - 10.3390/medicina58070846 [doi] LID - 846.* (1648-9144 (Electronic)).

125.	Lin, Y.F., et al., *Disseminated Mycobacterium chimaera infection in a patient with adult-onset immunodeficiency syndrome: case report.* (1471-2334 (Electronic)).

126.	Kajeekul, R.A.-O., et al., *Francisella sp., a Close Relative of Francisella orientalis, Causing Septicemia with Cholestatic Hepatitis in a Patient with Anti-Interferon-γ (IFN-γ) Autoantibodies. LID - 10.3390/tropicalmed7020025 [doi] LID - 25.* (2414-6366 (Electronic)).

127.	Hirayama, K., et al., *Disseminated Mycolicibacter arupensis and Mycobacterium avium co-infection in a patient with anti-interferon-γ neutralizing autoantibody-associated immunodeficiency syndrome.* (1437-7780 (Electronic)).

128.	Hidekawa, C., et al., *Anti-interferon-γ Antibody-seropositive Disseminated Nontuberculous Mycobacterial Infection Mimicking POEMS and TAFRO Syndromes.* (1349-7235 (Electronic)).

129.	de Castro, M.V., et al., *The oldest unvaccinated Covid-19 survivors in South America.* (1742-4933 (Print)).

130.	Boyle, S.A.-O., et al., *The unique diagnostic and management challenge of a patient with concomitant anti-interferon-gamma autoantibody associated immunodeficiency syndrome, IgG4-related disease, and treatment refractory, disseminated mycobacterium avium complex infection.* (1710-1484 (Print)).

131.	Zeitler, K., et al., *Recurrent disseminated Mycobacterium avium in a female patient from Thailand with anti-interferon-gamma autoantibodies: dilemma on treatment approach. LID - 10.1136/bcr-2020-237909 [doi] LID - e237909.* (1757-790X (Electronic)).

132.	Yang, C.H., F.C. Kuo, and C.H. Lee, *Pathological humerus fracture due to anti-interferon-gamma autoantibodies: A case report.* (2218-5836 (Print)).

133.	Uno, S.A.-O., et al., *R-CHOP Chemotherapy for Disseminated Mycobacterium avium Complex Disease due to Anti-Interferon-Gamma Autoantibodies: A Case Report.* (2328-8957 (Print)).

134.	Thapa, P.A.-O., et al., *Sweet Syndrome precipitated by Mycobacterium abscessus in a Laotian Man with Autoantibodies to Interferon Gamma.* (2324-7096 (Electronic)).

135.	Rocco, J.M., et al., *Bortezomib treatment for refractory nontuberculous mycobacterial infection in the setting of interferon gamma autoantibodies.* (2589-9090 (Electronic)).

136.	Peng, S.Y., et al., *Anti-IFN-γ Immunodeficiency Syndrome Presenting as Blurred Vision and Salmonellosis.* (1573-2592 (Electronic)).

137.	Ochoa, S., et al., *Daratumumab (Anti-CD38) for Treatment of Disseminated Nontuberculous Mycobacteria in a Patient With Anti-Interferon-γ Autoantibodies.* (1537-6591 (Electronic)).

138.	Mochizuka, Y., et al., *Endobronchial Lesions from Disseminated Mycobacterium avium Infection in a Patient with Anti-interferon-gamma Autoantibodies.* (1349-7235 (Electronic)).

139.	King, E.A.-O., V.K. Weaver, and M.H. Kestler, *Treatment Dilemmas in Disseminated Nontuberculous Mycobacterial Infections With Interferon-gamma Autoantibodies.* (2328-8957 (Print)).

140.	Jin, W.A.-O., et al., *Coinfection by Talaromyces marneffei and Mycobacterium abscessus in a human immunodeficiency virus-negative patient with anti-interferon-γ autoantibody: a case report.* (1473-2300 (Electronic)).

141.	Dahl, V.N., et al., *Disseminated Mycobacterium avium complex infection in a woman with anti-interferon-γ autoantibodies.* (2214-2509 (Print)).

142.	Asako, M., et al., *Mycobacterial lymphadenitis without granuloma formation in a patient with anti-interferon-gamma antibodies.* (1865-3774 (Electronic)).

143.	Ansari, R., et al., *Primary and Acquired Immunodeficiencies Associated With Severe Varicella-Zoster Virus Infections.* (1537-6591 (Electronic)).

144.	Yamaba, Y., et al., *Disseminated Mycobacterium avium Infection Complicated with Chylous Ascites in a Patient with Neutralizing Autoantibodies to Interferon-γ.* (1349-7235 (Electronic)).

145.	Yaghnam, I., et al., *Acquired tracheoesophageal fistula in disseminated Mycobacterium avium complex associated with anti-interferon-gamma autoantibodies. LID - 10.1136/bcr-2020-235661 [doi] LID - e235661.* (1757-790X (Electronic)).

146.	Weng, T.P., L.S. Syue, and N.Y. Lee, *Disseminated Mycobacterium szulgai infection in a patient with anti-interferon-gamma autoantibodies.* (2214-2509 (Print)).

147.	Rujirachun, P., J. Sangwongwanich, and M.A.-O. Chayakulkeeree, *Triple infection with Cryptococcus, varicella-zoster virus, and Mycobacterium abscessus in a patient with anti-interferon-gamma autoantibodies: a case report.* (1471-2334 (Electronic)).

148.	Roerden, M.A.-O., et al., *Simultaneous disseminated infections with intracellular pathogens: an intriguing case report of adult-onset immunodeficiency with anti-interferon-gamma autoantibodies.* (1471-2334 (Electronic)).

149.	Liang, X., et al., *Talaromyces marneffei infection relapse presenting as osteolytic destruction followed by suspected nontuberculous mycobacterium infection during 6 years of follow-up: A case update.* International Journal of Infectious Diseases, 2020. **93**: p. 208-210.

150.	Liang, X.N., et al., *Non-tuberculous mycobacterial infection and reactive dermatosis associated with adult-onset immunodeficiency due to anti-interferon-gamma autoantibodies: A case report.* (1536-5964 (Electronic)).

151.	Keragala, B., et al., *Disseminated Mycobacterium simiae infection in a patient with adult-onset immunodeficiency due to anti-interferon-gamma antibodies - a case report.* (1471-2334 (Electronic)).

152.	Yerramilli, A.A.-O., et al., *Disseminated Nontuberculous Mycobacterial Infection Associated With Acquired Immunodeficiency Due to Anti-Interferon γ Autoantibodies.* (2328-8957 (Print)).

153.	Yeh, Y.K., et al., *Disseminated Mycobacterium avium complex infection mimicking malignancy in a patient with anti-IFN-γ autoantibodies: a case report.* (1471-2334 (Electronic)).

154.	Xu, X., et al., *Chronic Mycobacterium avium skin and soft tissue infection complicated with scalp osteomyelitis possibly secondary to anti-interferon-γ autoantibody formation.* (1471-2334 (Electronic)).

155.	Wongkamhla, T., P. Chongtrakool, and A.A.-O.X. Jitmuang, *A case report of Talaromyces marneffei Oro-pharyngo-laryngitis: a rare manifestation of Talaromycosis.* (1471-2334 (Electronic)).

156.	Wen and P., *Severe disseminated*

*Mycobacterium avium complex infection in a pregnant woman with*

*anti-interferon-γ autoantibody*, M. Wei, Xu, Y., & Wang, M., Editor. (2019).

157.	Tanaka, S.A.-O., et al., *Pathogenicity of Mycolicibacterium phlei, a non-pathogenic nontuberculous mycobacterium in an immunocompetent host carrying anti-interferon gamma autoantibodies: a case report.* (1471-2334 (Electronic)).

158.	Su, S.S., et al., *Disseminated Talaromyces marneffei And Mycobacterium avium Infection Accompanied Sweet's Syndrome In A Patient With Anti-Interferon-γ Autoantibodies: A Case Report.* (1178-6973 (Print)).

159.	Namkoong, H., et al., *First report of hepatobiliary Mycobacterium avium infection developing obstructive jaundice in a patient with neutralizing anti-interferon-gamma autoantibodies.* (2052-2975 (Print)).

160.	Liew, W.K., et al., *Juvenile-Onset Immunodeficiency Secondary to Anti-Interferon-Gamma Autoantibodies.* (1573-2592 (Electronic)).

161.	Koizumi, Y.A.-O., et al., *Chylous ascites, anti-interferon-gamma autoantibody, and angioimmunoblastic T-cell lymphoma: a rare but intriguing connection over Mycobacterium avium.* (1432-1831 (Electronic)).

162.	Kashihara, E., et al., *Case Report: Disseminated Mycobacterium kansasii Disease in a Patient with Anti-Interferon-Gamma Antibody.* (1476-1645 (Electronic)).

163.	Cheng, A., et al., *Disseminated Mycobacterium abscessus Infections in Patients with Neutralizing Anti-interferon-gamma Autoantibodies Treated with Rifabutin-based Combination Regimens.* (2328-8957 (Electronic)).

164.	Xu, H., et al., *Sweet's Syndrome Associated with Talaromyces marneffei and Mycobacterium abscessus Infection Due to Anti-interferon-gamma Autoantibodies.* (1998-3611 (Electronic)).

165.	Miyashita, K., et al., *Disseminated Mycobacterium avium Infection Presenting with Bladder Lesions in a Patient with Interferon-γ-neutralizing Autoantibodies.* (1349-7235 (Electronic)).

166.	Hung, T.C., et al., *Anti-interferon-γ auto-anti-body and disseminated non-tuberculous mycobacteria infections in thyroid cancer: a case report.* QJM: An International Journal of Medicine, 2018. **111**(9): p. 647-648.

167.	Wu, U.I., et al., *Neutralizing antiinterferon-γ autoantibodies causing disseminated Mycobacterium avium complex infection in an HIV-infected patient on successful combination antiretroviral therapy.* (1473-5571 (Electronic)).

168.	van de Vosse, E.A.-O., et al., *A 38-year-old woman with necrotising cervical lymphadenitis due to Histoplasma capsulatum.* (1439-0973 (Electronic)).

169.	Li, W.S., et al., *Osteolytic lesions resulting from opportunistic infections.* (2410-8650 (Electronic)).

170.	Koizumi, Y.A.-O., et al., *Rituximab Restores IFN-γ-STAT1 Function and Ameliorates Disseminated Mycobacterium avium Infection in a Patient with Anti-Interferon-γ Autoantibody.* (1573-2592 (Electronic)).

171.	Furuya, H., et al., *SAPHO syndrome-like presentation of disseminated nontuberculous mycobacterial infection in a case with neutralizing anti-IFNγ autoantibody.* (1462-0332 (Electronic)).

172.	Chang, P.H. and Y.C. Chuang, *Anti-interferon-γ autoantibody-associated disseminated Mycobacterium abscessus infection mimicking parotid cancer with multiple metastases: A case report.* (1536-5964 (Electronic)).

173.	Asakura T Fau - Namkoong, H., et al., *Disseminated Mycobacterium genavense Infection in Patient with Adult-Onset Immunodeficiency.* (1080-6059 (Electronic)).

174.	Xie, Y.L., et al., *Severe Paradoxical Reaction During Treatment of Disseminated Tuberculosis in a Patient With Neutralizing Anti-IFNγ Autoantibodies.* (1537-6591 (Electronic)).

175.	Tham, E.H., et al., *Neutralizing Anti-Interferon-Gamma Autoantibody Levels May Not Correlate With Clinical Course of Disease.* Clinical Infectious Diseases, 2016. **63**(4): p. 572-573.

176.	Valour F Fau - Perpoint, T., et al., *Interferon-γ Autoantibodies as Predisposing Factor for Nontuberculous Mycobacterial Infection.* (1080-6059 (Electronic)).

177.	Pruetpongpun, N., et al., *Disseminated Talaromyces marneffei and Mycobacterium abscessus in a Patient With Anti-Interferon-γ Autoantibodies.* (2328-8957 (Print)).

178.	Noguchi, S., et al., *A Case of Disseminated Mycobacterium avium Complex Infection Mimicking Systemic Metastases From Lung Cancer.* Journal of Thoracic Imaging, 2016. **31**(6).

179.	Liu, T.T., et al., *Nontuberculous mycobacterial infection with concurrent IgG4-related lymphadenopathy.* (1600-0463 (Electronic)).

180.	Lee, T.A.-O., et al., *Disseminated nontuberculous mycobacterial infection with multifocal retinitis and vasculitis in an immunocompromised patient with anti-IFN-ɣ autoantibodies.* (1869-5760 (Print)).

181.	Ikeda, H., et al., *Severe Disseminated Mycobacterium avium Infection in a Patient with a Positive Serum Autoantibody to Interferon-γ.* (1349-7235 (Electronic)).

182.	Otome, O., M. O'Reilly, and L. Lim, *Disseminated Mycobacterium haemophilum skeletal disease in a patient with interferon-gamma deficiency.* (1445-5994 (Electronic)).

183.	Nishimura, T., et al., *Recurrence of disseminated Mycobacterium avium complex disease in a patient with anti-gamma interferon autoantibodies by reinfection.* (1098-660X (Electronic)).

184.	Hanitsch, L.G., et al., *Late-Onset Disseminated Mycobacterium avium intracellulare Complex Infection (MAC), Cerebral Toxoplasmosis and Salmonella Sepsis in a German Caucasian Patient with Unusual Anti-Interferon-Gamma IgG1 Autoantibodies.* (1573-2592 (Electronic)).

185.	Wang, H.H., et al., *Subcutaneous Sweet syndrome associated with disseminated nontuberculous mycobacterial infection leading to the diagnosis of adult-onset immunodeficiency.* (1097-6787 (Electronic)).

186.	Suzuki, K., et al., *Pulmonary Mycobacterium fortuitum infection with cervical lymphadenitis in a patient carrying autoantibodies to interferon-γ.* (1349-7235 (Electronic)).

187.	O'Connell, E., et al., *The first US domestic report of disseminated Mycobacterium avium complex and anti-interferon-γ autoantibodies.* (1573-2592 (Electronic)).

188.	DeLeon, T.T., et al., *Mycobacterium avium complex empyema in a patient with interferon gamma autoantibodies.* (2165-8242 (Electronic)).

189.	Czaja, C.A., et al., *Rituximab as successful adjunct treatment in a patient with disseminated nontuberculous mycobacterial infection due to acquired anti-interferon-γ autoantibody.* (1537-6591 (Electronic)).

190.	Chan, J.F., et al., *Adult-onset immunodeficiency due to anti-interferon-gamma autoantibody in mainland Chinese.* (2542-5641 (Electronic)).

191.	Yamaguchi, E., et al., *A case of disseminated nontuberculous mycobacteriosis and cerebellar toxoplasmosis with autoantibody to interferon-γ.* Sarcoidosis, vasculitis, and diffuse lung diseases : official journal of WASOG, 2013. **30 4**: p. 312-6.

192.	Watanabe, T., et al., *Disseminated <em>Mycobacterium avium</em> complex infection in a patient with anti-interferon-gamma autoantibody.* European Journal of Internal Medicine, 2013. **24**: p. e202.

193.	Poulin, S., et al., *Fatal Mycobacterium colombiense/cytomegalovirus coinfection associated with acquired immunodeficiency due to autoantibodies against interferon gamma: a case report.* (1471-2334 (Electronic)).

194.	Nei, T., et al., *A non-HIV case with disseminated Mycobacterium kansasii disease associated with strong neutralizing autoantibody to interferon-γ.* (2213-0071 (Print)).

195.	Chaisathaphol, T. and A. Jitmuang, *Disseminated Mycobacterium avium and recurrent Salmonella group D infection in a patient with autoantibodies to interferon-gamma.* (0125-1562 (Print)).

196.	Tang, B.S., et al., *Disseminated penicilliosis, recurrent bacteremic nontyphoidal salmonellosis, and burkholderiosis associated with acquired immunodeficiency due to autoantibody against gamma interferon.* (1556-679X (Electronic)).

197.	Koya, T., et al., *Anti-interferon-gamma autoantibody in a patient with disseminated Mycobacterium avium complex.* (1341-321X (Print)).

198.	Baerlecken, N., et al., *Recurrent, multifocal Mycobacterium avium-intercellulare infection in a patient with interferon-gamma autoantibody.* (1537-6591 (Electronic)).

199.	Kampmann, B., et al., *Acquired predisposition to mycobacterial disease due to autoantibodies to IFN-gamma.* (0021-9738 (Print)).

200.	Höflich, C., et al., *Naturally occurring anti-IFN-gamma autoantibody and severe infections with Mycobacterium cheloneae and Burkholderia cocovenenans.* (0006-4971 (Print)).

201.	Döffinger, R., et al., *Autoantibodies to interferon-gamma in a patient with selective susceptibility to mycobacterial infection and organ-specific autoimmunity.* (1537-6591 (Electronic)).

202.	Pozzetto B Fau - Mogensen, K.E., et al., *Characteristics of autoantibodies to human interferon in a patient with varicella-zoster disease.* (0022-1899 (Print)).
